# The Correlation of Mutations and Expressions of Genes within the PI3K/Akt/mTOR Pathway in Breast Cancer—A Preliminary Study

**DOI:** 10.3390/ijms22042061

**Published:** 2021-02-19

**Authors:** Przemysław Kołodziej, Marcin Nicoś, Paweł A. Krawczyk, Jacek Bogucki, Agnieszka Karczmarczyk, Daniel Zalewski, Tomasz Kubrak, Elżbieta Kołodziej, Anna Makuch-Kocka, Barbara Madej-Czerwonka, Bartosz J. Płachno, Janusz Kocki, Anna Bogucka-Kocka

**Affiliations:** 1 Chair and Department of Biology and Genetics, Medical University of Lublin, 20-093 Lublin, Poland; daniel.zalewski@umlub.pl; 2 Department of Pneumonology, Oncology and Allergology, Medical University of Lublin, 20-954 Lublin, Poland; marcin_nicos@interia.pl (M.N.); pawel.krawczyk@umlub.pl (P.A.K.); 3 Department of Organic Chemistry, Medical University of Lublin, 20-093 Lublin, Poland; jacek.bogucki@umlub.pl; 4 Department of Experimental Haematooncology, Medical University of Lublin, 20-093 Lublin, Poland; agnieszka.piechnik@umlub.pl; 5 Department of Biochemistry and General Chemistry, Faculty of Medicine, University of Rzeszow, 35-310 Rzeszów, Poland; kubrak.tomasz@gmail.com; 6 Department of Clinical Genetics, Medical University of Lublin, 20-080 Lublin, Poland; elzbieta.kolodziej@umlub.pl (E.K.); janusz.kocki@umlub.pl (J.K.); 7 Department of Pharmacology, Medical University of Lublin, 20-059 Lublin, Poland; anna.makuch-kocka@umlub.pl; 8 Department of Breast Surgery, District Specialist Hospital of Stefan Cardinal Wyszynski in Lublin, 20-718 Lublin, Poland; barbara.madej-czerwonka@umlub.pl; 9 Department of Human Anatomy, Medical University of Lublin, 20-090 Lublin, Poland; 10 Department of Plant Cytology and Embryology, Institute of Botany, Faculty of Biology, Jagiellonian University in Kraków, 30-387 Kraków, Poland; bartosz.plachno@uj.edu.pl

**Keywords:** PI3K/Akt/mTOR pathway, breast cancer, mutations, expressions of genes, biomarkers, therapeutic targets

## Abstract

There is an urgent need to seek new molecular biomarkers helpful in diagnosing and treating breast cancer. In this elaboration, we performed a molecular analysis of mutations and expression of genes within the PI3K/Akt/mTOR pathway in patients with ductal breast cancer of various malignancy levels. We recognized significant correlations between the expression levels of the studied genes. We also performed a bioinformatics analysis of the data available on the international database TCGA and compared them with our own research. Studies on mutations and expression of genes were conducted using High-Resolution Melt PCR (HRM-PCR), Allele-Specific-quantitative PCR (ASP-qPCR), Real-Time PCR molecular methods in a group of women with ductal breast cancer. Bioinformatics analysis was carried out using web source Ualcan and bc-GenExMiner. In the studied group of women, it was observed that the prevalence of mutations in the studied *PIK3CA* and *AKT1* genes was 29.63%. It was stated that the average expression level of the *PIK3CA*, *PIK3R1*, *PTEN* genes in the group of breast cancer patients is lower in comparison to the control group, while the average expression level of the *AKT1* and *mTOR* genes in the studied group was higher in comparison to the control group. It was also indicated that in the group of patients with mutations in the area of the *PIK3CA* and *AKT1* genes, the *PIK3CA* gene expression level is statistically significantly lower than in the group without mutations. According to our knowledge, we demonstrate, for the first time, that there is a very strong positive correlation between the levels of *AKT1* and *mTOR* gene expression in the case of patients with mutations and without mutations.

## 1. Introduction

The PI3K/Akt/mTOR pathway plays an important role in the regulation of the cells’ cycle; it impacts the development and differentiation of cells [1,2,3,4,5,6,7]. The PI3K/Akt/mTOR signaling pathway plays an important role in the processes of tumorigenesis. Gene mutations within the PI3K/Akt/mTOR pathway are identified in numerous types of malignancies, among others, lung cancer, brain cancer, colorectal cancer, ovarian cancer, breast cancer and uterus cancer [3,8,9,10,11,12,13,14,15].

Currently, numerous studies have been conducted, with the purpose to obtain profound knowledge on the function of the PI3K/Akt/mTOR pathway and its role in cancer cells. This will allow for the introduction of new treatment plans with the application of PI3K, Akt and mTOR kinase inhibitors, which constitute the main elements of the pathway. Multiple inhibitors of the PI3K/Akt/mTOR pathway are in preclinical or clinical trials. There are promising data indicating that rapalogs or inhibitors of PI3K/Akt are active in breast cancers [6,16,17,18,19,20]. In cancer treatment, various therapeutic procedures are used, including polytherapies. In the treatment of breast cancer, PI3K, mTOR Act kinase inhibitors are also used with increasing frequency; therefore, we compared the level of gene expression associated with the PI3K/Akt/mTOR pathway with and without mutations in breast cancer patients.

The association concerning the presence of mutations and changes in the expression of genes within the PI3K/Akt/mTOR pathway, as well as their impact on breast cancer prognosis, have been evaluated by many studies. However, the results of these studies are still inconclusive, i.e., some studies showed favorable impacts, some studies showed adverse impacts and other papers did not show any prognostic significance [4,21,22,23,24,25,26,27,28,29,30,31,32,33].

In order to extend the knowledge, we assessed the prevalence of the E542K, E545K, H1047R mutations within the *PIK3CA* gene and the E17K mutation within the *AKT1* gene, and the expression of the *PIK3CA*, *PIK3R1*, *PTEN*, *AKT1*, *mTOR* genes in a cohort of patients with breast cancer. We evaluated the relation between the studied features and patients’ age, and the histological malignancy level of tumors, lymph node metastasis, ER, PR, HER2 status and Ki-67 index were determined. Moreover, we performed a bioinformatics analysis of the data available on the international database TCGA and compared them with our own research.

## 2. Results

### 2.1. Results of the Mutation Analysis of Selected Genes

Using PCR-based techniques described in the Methods Section, we identified gene mutations related to the PI3K/Akt/mTOR pathway in 29.63% (16/54) of breast cancer patients.

The *PIK3CA* gene mutations in exon 9 were in 5.56% (3/54) of the studied cohort-1.85% and 3.7% were distinguished as E542K (c.1624G>A) and E545K (c.1633G>A) substitutions, respectively. Moreover, substitution H1047R (c.3140A>G) in exon 20 of *PIK3CA* gene was observed in 18.52% (10/54) of patients. While the E17K (c.49G>A) substitution in exon 4 of the *AKT1* gene was observed in 7.4% (4/54) of patients (Table 1). In one patient, both the E545K mutation in the *PIK3CA* gene and the E17K mutation in the *AKT1* gene were observed simultaneously.

The results of our study indicated that, in the group of female patients in whom mutations in the *PIK3CA* and *AKT1* genes were observed, the Ki-67 index was significantly lower than in the group of female patients without mutations (*p* = 0.020). Moreover, the frequency of detected mutations does not depend on the patients’ age, lymph node metastasis, ER, PR or HER2 status and Bloom Richardson Scale (Table 2, Table S1).

### 2.2. Results of the Expression Analysis of Selected Genes

#### 2.2.1. The Expression Level of Studied Genes—Comparison between Our Results and Those Contained in the TCGA Database

**Figure 1:**
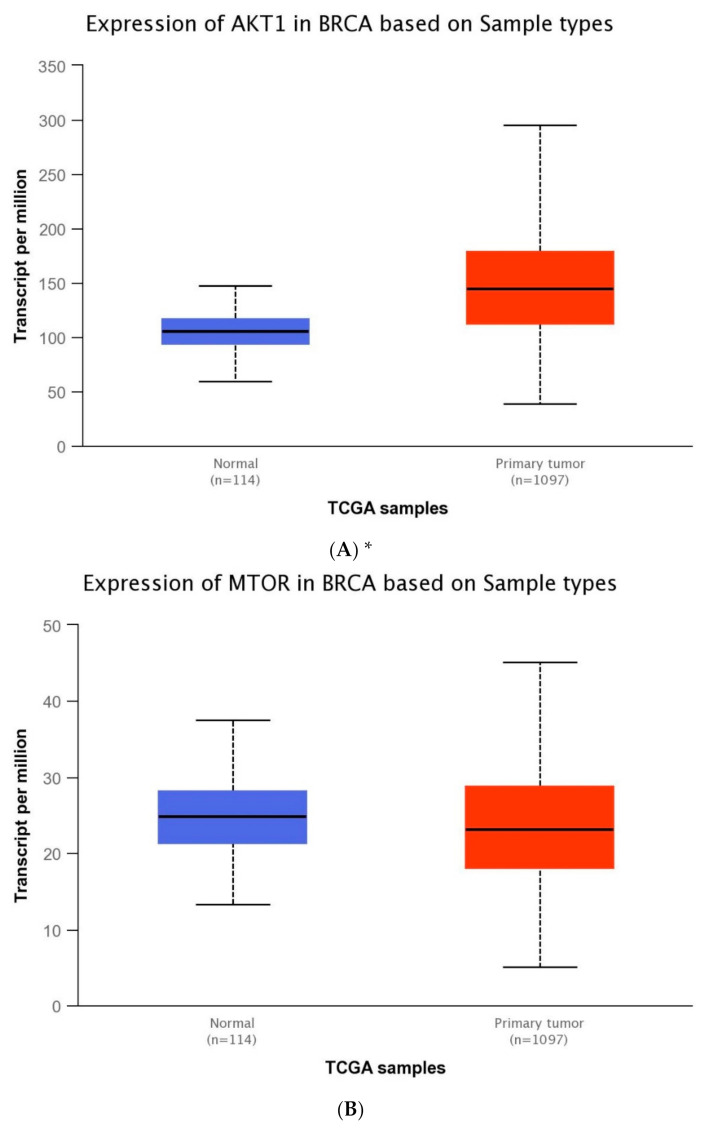

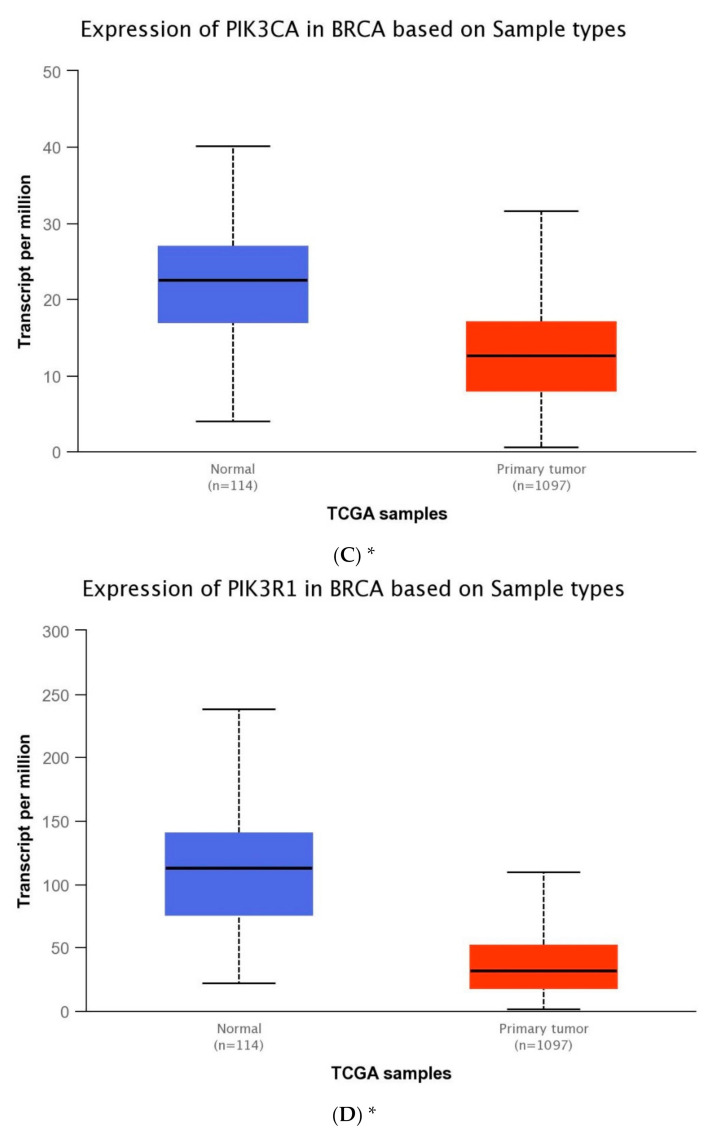

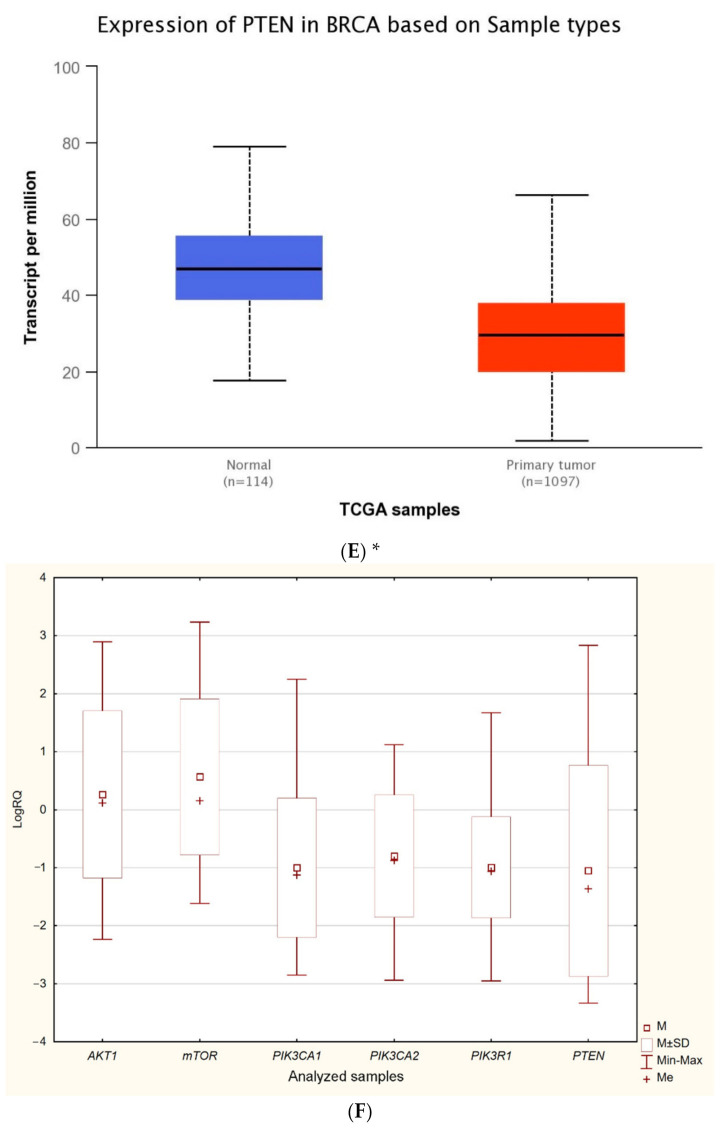
Differences between the expression level of the genes within the PI3K/Akt/mTOR pathway in normal tissues and breast cancer in the TCGA samples: *AKT1* (**A**), *MTO*R (**B**), *PIK3C*A (**C**), *PIK3R1* (**D**), *PTEN* (**E**) (web source the Ualcan) and in analyzed samples (**F**) * *p* < 0.05.

The expression level of five genes: *PIK3CA* (1. exon 20–21, 2. exon 9–10) *PIK3R1*, *PTEN*, *AKT1*, *mTOR,* was obtained using the real-time PCR technique in 54 breast cancer patients. It was indicated that the average expression level of the *PIK3CA*, *PIK3R1*, *PTEN* genes in the studied group was lower in comparison to the control group; while the average expression level of the *AKT1* and *mTOR* genes was higher in the studied group in comparison to the control group (Figure 1F). Data contained in the TCGA database show the results of studies on the expression of genes tested using the sequencing method. The analysis of the data contained in TCGA confirmed a lower level of *PIK3CA, PIK3R1* and *PTEN* gene expression in breast cancer tissues compared to normal tissues and a higher level of the *AKT1* gene. The expression level of the *mTOR* gene between the neoplastic and normal tissue did not differ (Figure 1A–E). Accurate statistical significance located in the Table S2.

#### 2.2.2. The Expression Level of Studied Genes Depending on the Presence of Mutations

**Figure 2:**
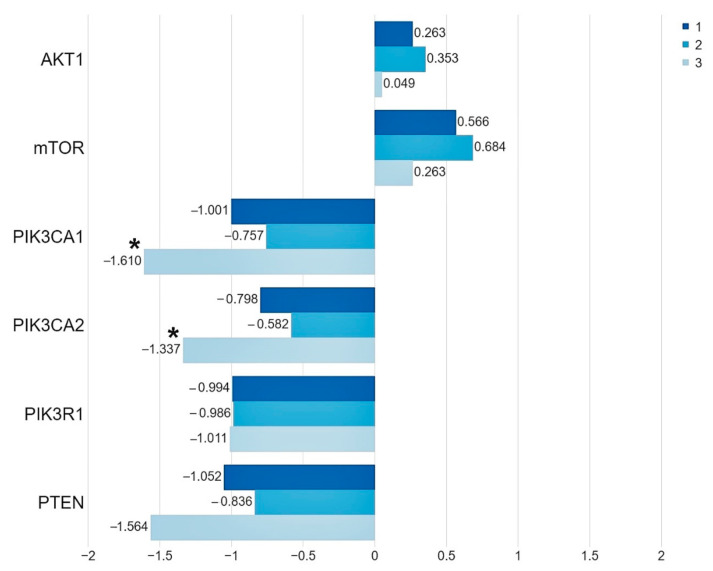
The mean expression level (expressed in logRQ) of the studied genes related to the PI3K/Akt/mTOR pathway in the: (1) entire studied group; (2) in the group of patients without mutations; (3) in the group of patients with mutations * *p* < 0.05.

We demonstrated that, in the group of patients harboring the studied mutations in the *PIK3CA* and *AKT1* genes, the level of the *PIK3CA* gene expression was significantly lower than in the group of patients without mutations (*PIK3CA* 1. *p* = 0.006, 2. *p* = 0.034). While the expression level of the *PTEN*, *PIK3R1*, *AKT1* and *mTOR* genes does not depend on the presence of mutations (Figure 2, Table 3, Table S3).

**Figure 3:**
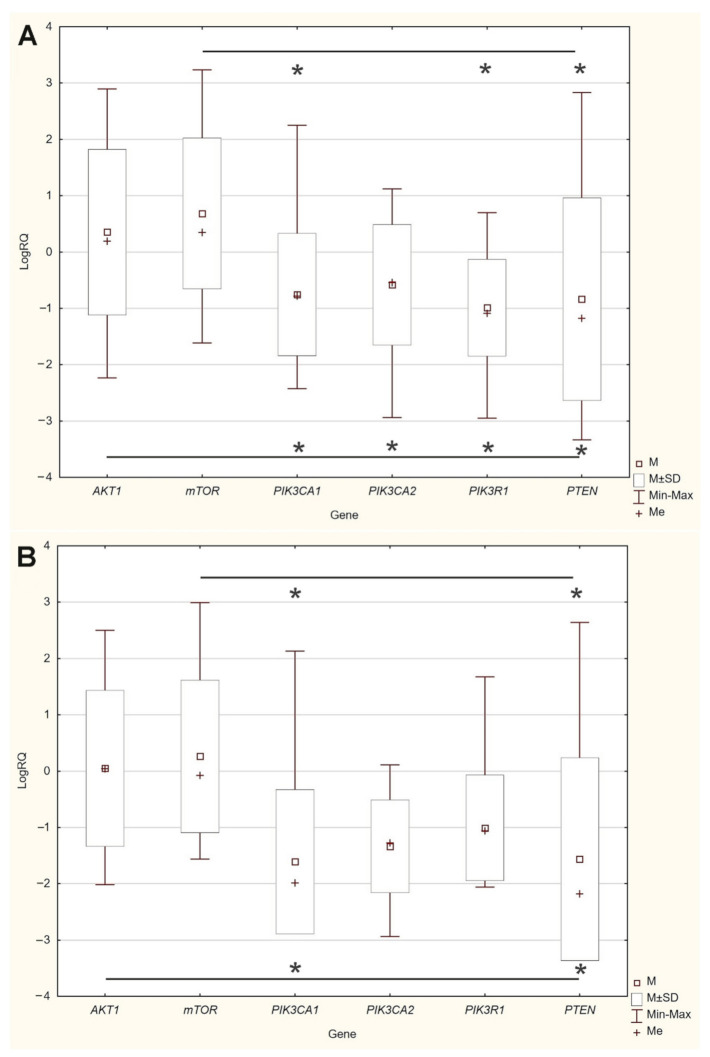
Differences between the expression level of the studied genes: (**A**) in the group of patients without mutations; (**B**) in the group of patients with mutations * *p* < 0.05.

An analysis of the differences between the expression levels of the studied genes showed statistically significant differences between the *AKT1*, *mTOR* gene expression levels and the *PIK3CA*, *PIK3R1*, *PTEN* gene expression levels in the group of patients without mutations and with mutations (Figure 3).

#### 2.2.3. The Expression Level of the Studied Genes Depending on Clinical Parameters—Comparison between Our Results and Those Contained in the TCGA Database

**Figure 4:**
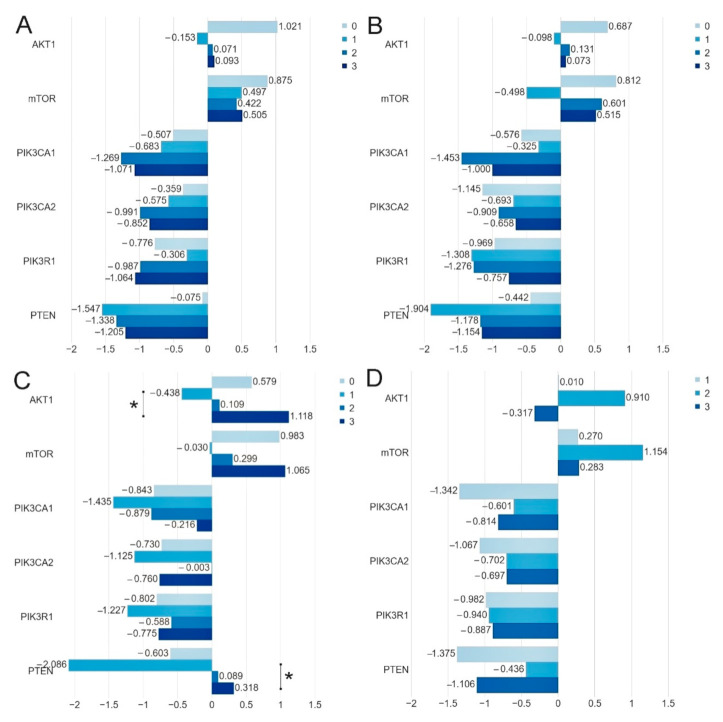
Comparison of the mean expression level (expressed in logRQ) of the studied genes depending on the: (**A**) ER-0 (−), 1 (1+), 2 (2+), 3 (3+); (**B**) PR-0 (−), 1 (1+), 2 (2+), 3 (3+); (**C**) HER2-0 (−), 1 (1+), 2 (2+), 3 (3+) status and (**D**) Ki-67 index-1 (≤10%), 2 (>10%-≤ 50%), 3 (>50%-≤90%) in the entire study group * *p* < 0.05.

The results of our study indicated that the expression levels of the *AKT1* (*p* = 0.0377) and *PTEN* (*p* = 0.0111) genes do depend on the HER2 status. The expression level of the *PIK3CA*, *PIK3R1* and *mTOR* genes does not depend on the HER2 status. It was also found that the expression levels of the *AKT1* (*p* = 0.0317), *mTOR* (0.0154) and *PTEN* (*p* = 0.0202) genes do depend on the Bloom Richardson Scale. Moreover, it was stated that the expression level of the *PIK3CA*, *PIK3R1*, *PTEN*, *AKT1*, *mTOR* genes does not depend on the patients’ age, lymph node metastases, ER, PR status and Ki-67 index (Table 4, Figure 4, Tables S4–S9).

Using the bc-GenExMiner web source, we analyzed the expression of the studied genes depending on the clinical parameters (age, ER, PR, HER2 and node status). Analysis of data in the TCGA database showed statistically significant differences between the expression of selected genes and with the following clinical parameters: ER status-*AKT1* (*p* = 0.0004), *PIK3CA* (*p* = 0.0285), *PIK3R1* (*p* = 0.0039) and *PTEN* (<0.0001); PR status-*PIK3R1*(<0.0001), *PTEN*(<0.0001); HER2 status-*AKT1* (*p* = 0.0002), *PTEN* (*p* = 0.0227) nodes status-*PIK3R1* (*p* = 0.0228) (Table 4, Figures S1–S5). Differences between our results and those contained in the TCGA database may result from a relatively small group of patients participating in our study. Differences may also result from the dissimilarity of the surveyed group (the population comes from Poland). Therefore, there is a need to confirm them in an independent cohort of patients.

The conducted analysis the data available on international database TCGA using the Kaplan–Meier Plotter (web source the Ualcan) showed that no clear relation between expression level of the studied genes and breast cancer patients survival-*AKT1 (p* = 0.44), *MTOR* (*p* = 0.79), *PIK3CA* (*p* = 0.14), *PIK3R1* (*p* = 0.48), *PTEN* (*p* = 0.25) (Figure S6).

#### 2.2.4. The Correlation between the Expression Levels of Studied Genes—Comparison between Our Results and Those Contained in the TCGA Database

**Figure 5:**
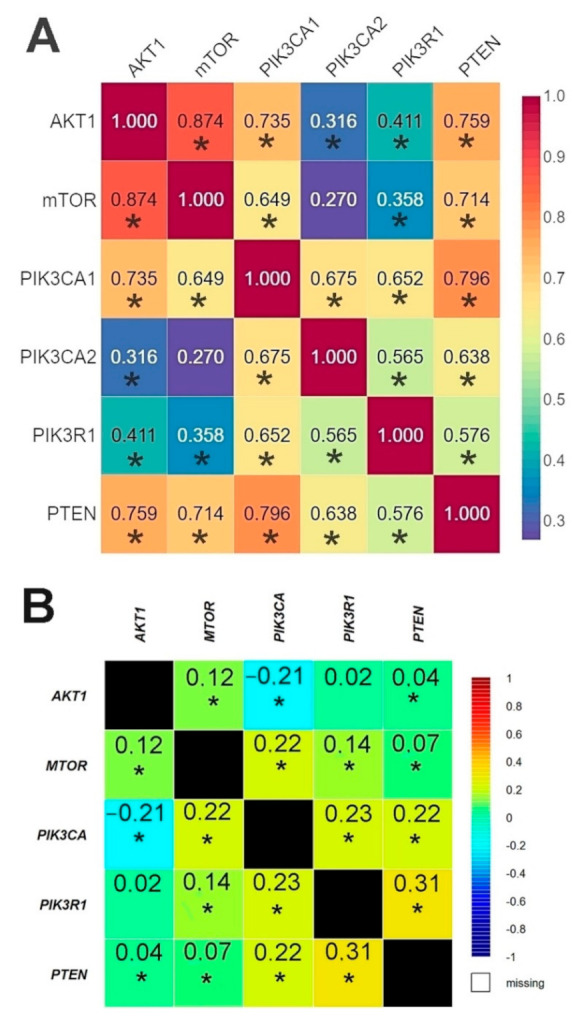
Comparison of the correlation between the expression level of the studied genes in the analyzed patients using Spearman’s rank correlation coefficient (**A**) and patients from TCGA database using Pearson’s correlation coefficient (**B**) (web source the bc-GenExMiner) * *p* < 0.05.

The results of the correlation analysis showed a statistically significant correlation between all genes except for *PIK3CA* (2) and *mTOR.* Using the bc-GenExMiner web source, we conducted a correlation analysis of the data available on the TCGA database between the studied genes. The analysis confirmed the existence of statistically significant correlations between all the studied genes except for *AKT1* and *PIK3R1* (Figure 5, Tables S10 and S11, Figures S7–S16).

**Figure 6:**
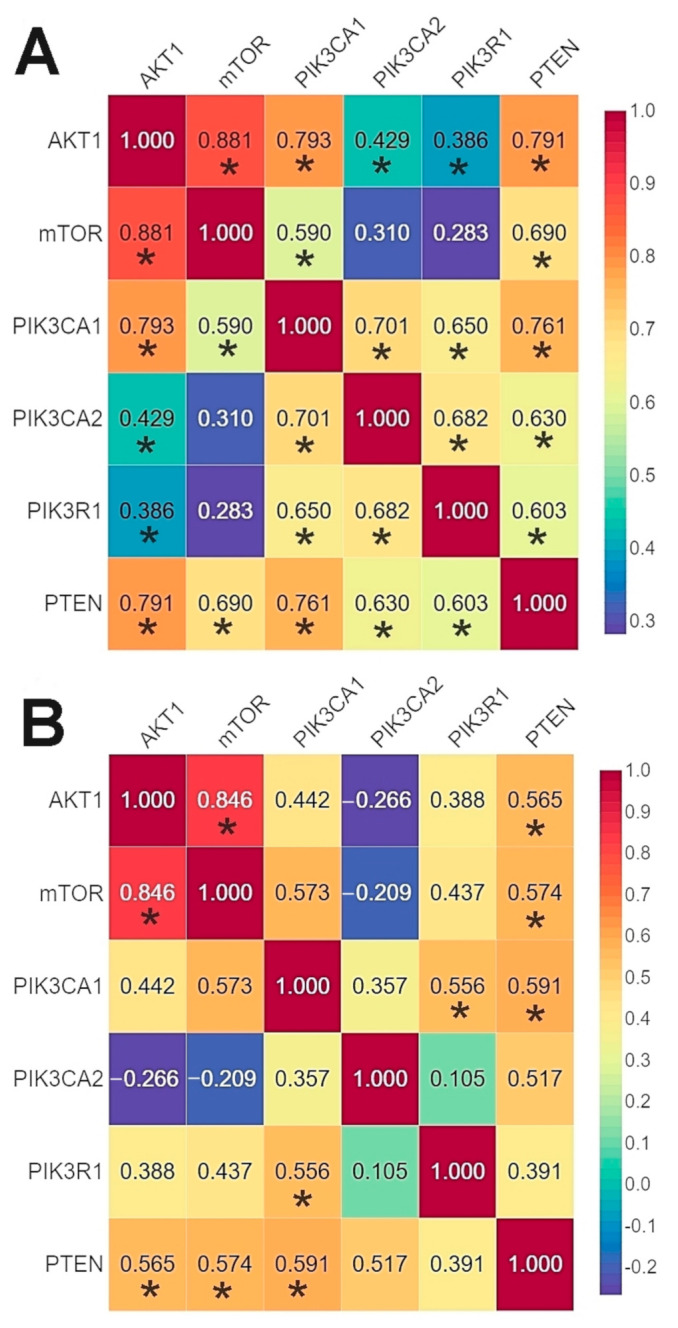
Correlation between the expression level of the studied genes using Spearman’s rank correlation coefficient: (**A**) in the group of patients without mutations; (**B**) in the group of patients with mutations * *p* < 0.05.

The r-Spearman analysis showed a high correlation between the expression of the *AKT1* and *mTOR* genes in the group of patients without mutations, and in the group of patients with mutations (Figure 6, Tables S12–S13).

**Figure 7:**
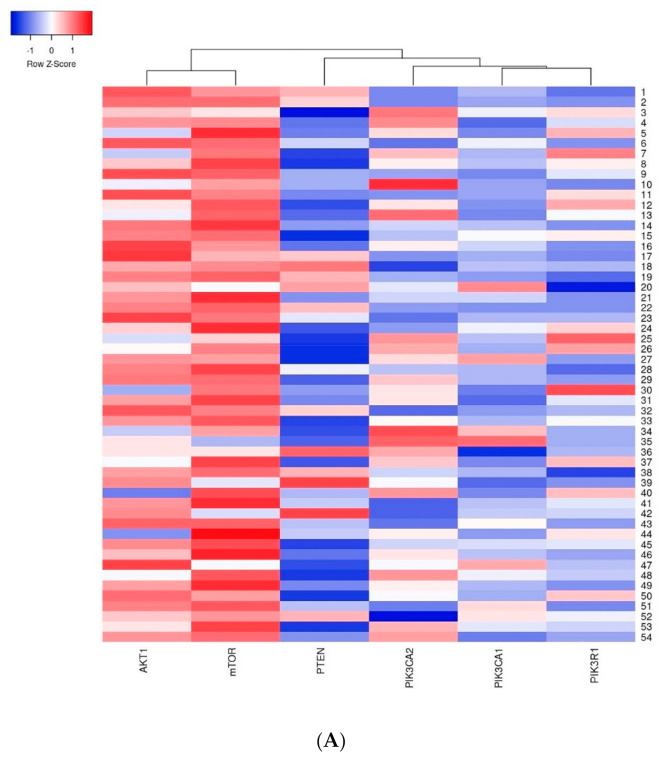

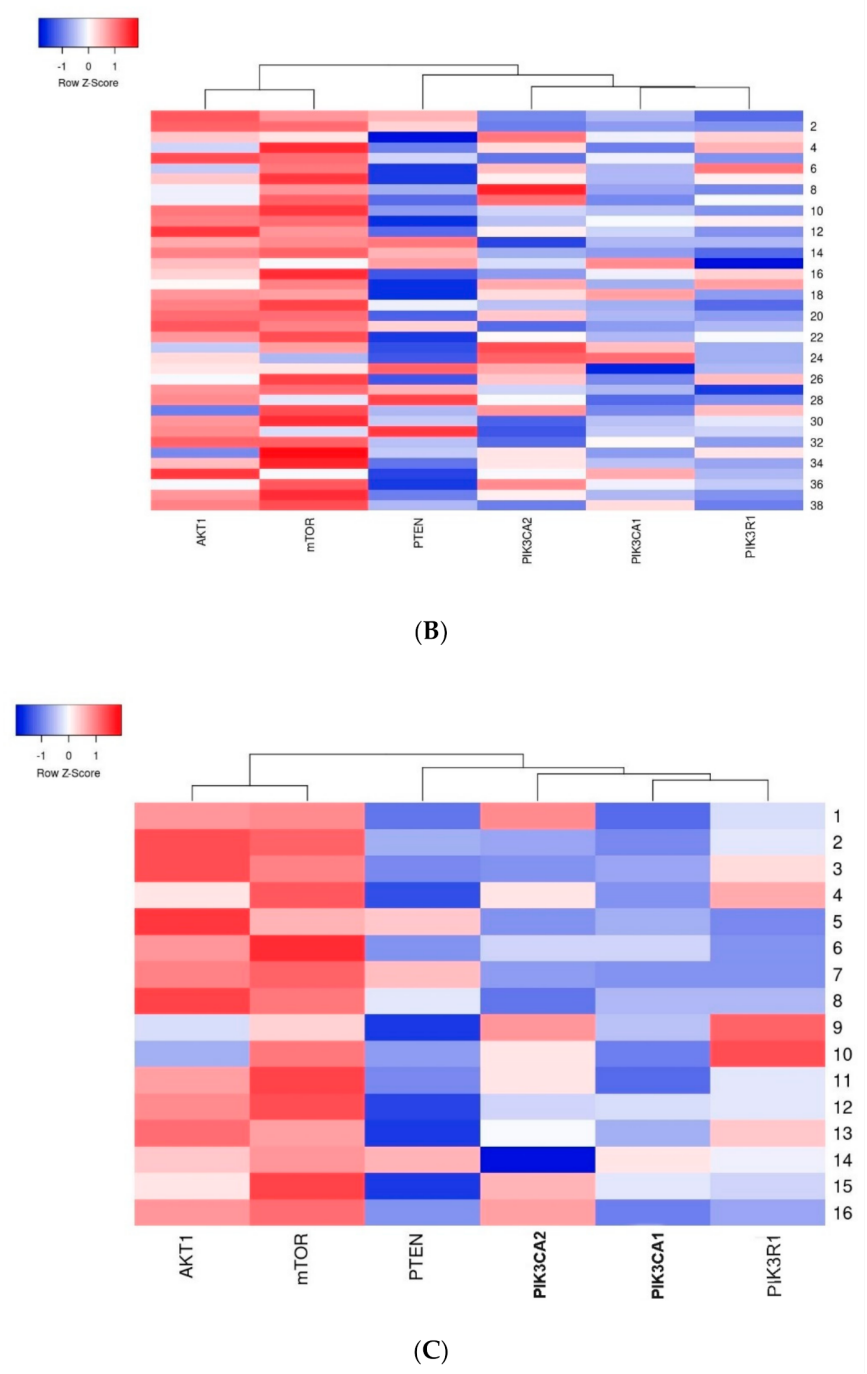
Heatmap presenting a clustering analysis of the expression level of the studied genes (clustering method: average linkage; Euclidean distance measurement method): (**A**) in the entire studied group; (**B**) in the group of patients without mutations; (**C**) in the group of patients with mutations.

The first group of genes which indicated a strong relation with one another included the *AKT1* and *mTOR* genes–a strong dependency between these genes was observed in all analyzed cases. The second group included the *PIK3CA*, *PIK3R1* and *PTEN* genes where the relation between the studied genes was weaker than in the first group (Figure 7).

## 3. Discussion

The conducted study allowed us to compare the clinical and molecular features, such as age, lymph node metastasis, ER, PR, HER2 status, Ki-67 index and the histopathological malignancy level with the mutations and expressions of genes related to the PI3K/Akt/mTOR pathway in breast cancer. Moreover, performed a bioinformatics analysis allowed us to compare the data available on the international database TCGA with our own research. *PIK3CA* and *AKT1* gene mutations are observed in numerous types of cancers, including breast cancer. The most frequent mutations in the *PIK3CA* gene occur in exon 9 and exon 20 (approx. 17–40%) [12,13,14,34,35,36,37,38]. Meanwhile, the most frequent mutation in the AKT1 gene (E17K substitution) is observed in approx. 4% of breast cancer patients [4,13,38,39,40,41]. The prevalence of *PIK3CA* (E542K, E545K, H1047R) and *AKT1* (E17K) mutations among our patients are compliant with the results obtained by authors conducting similar studies and they refer to 24% and 7.4%, respectively. Moreover, their prevalence does not depend on the patients’ age, lymph node metastasis and the level of histopathological malignancy of tumors. Additionally, in the literature, no clear relationship between *PIK3CA* mutations and lymph node metastasis or histopathological diagnosis was observed [25,36,42,43,44]. In most of the analyzed publications, a significant relationship between the prevalence of mutations and the expression of the ER and PR receptors was observed [4,45]. In our cohort, the detected mutations do not depend on the expression level of those receptors. In recent years, numerous papers dedicated to the expression of genes and proteins related to the PI3K/Akt/mTOR pathway in breast cancer have been created. Moreover, the relation between their expression and gene mutation was described [1,33,46,47,48,49,50,51,52,53,54]. Additionally, the results of our study indicated that in the group of female patients in whom mutations in *PIK3CA* (E542K, E545K, H1047R) and *AKT1* (E17K) genes were observed, the expression level of the *PIK3CA* gene was significantly lower than in the group of female patients without mutations. Moreover, the existence of a very high correlation between the expression levels of the *AKT1* and *mTOR* genes was also in that study for the first time. Palimaru et al. demonstrated that both *PIK3CA* and *PTEN* gene expressions were significantly increased in breast carcinoma tissue compared to normal breast tissue (*p* = 2 × 10^−11^) and (*p* < 0.001), respectively. *PIK3CA* mutations were present in 68 out of 175 patients (39%) but were not associated with *PIK3CA* expression (*p* = 0.59). Additionally, the expressions of *PIK3CA* and *PTEN* mRNA, and *PIK3CA* mutations in breast carcinomas were not associated with the presence of lymph node metastases [51]. Cizkova et al. presented the results of the analysis of the *PIK3CA*, *PIK3R1* and *AKT1* mRNA expression levels, which were assessed in the whole series of 458 samples. *PIK3R1* underexpression was found in 283 (61.8%) cases, indicating a relevant tumor alteration occurring in the majority of tumor samples. *PIK3CA* was deregulated in only a minority of tumor samples: overexpressed in 18 (3.9%) and underexpressed in 40 (8.7%) cases. *AKT1* overexpression was present in 116 (25.3%) of the 458 available samples. In the authors’ own studies–similarly to the studies conducted by Cizkova et al.–*PIK3R1* underexpression (46; 85.19% cases) and *AKT1* overexpression (29; 53.7% cases) was observed [52]. Kim JY et al. demonstrated that nine out of ten *PIK3CA* mutations occurred at known hotspots: E545, H1047, and G1049, without a relationship between the gene mutation and expression. Moreover, they did not find a correlation between the genetic mutation and expression of the *PIK3CB*, *PIK3CD, PIK3CG*, *PTEN* genes [33]. In a publication by Mutee et al., a significantly higher number of breast cancer tissues were found to express the mTOR protein in various grades of intensity as compared to normal breast tissues. However, there were no significant relationships between clinicopathological variables (age group, clinical stage and receptor status) and mTOR protein expression (*n* = 78 breast cancer tissues and *n* = 53 of normal breast tissues) [46]. Additionally, Cheng et al. demonstrated that among the 71 cases of breast cancer tissues, 54.9% were mTOR-positive and exhibited a significantly higher expression than the 32 cases of normal tissues (21.9%) [54]. We analyzed the data present on the PI3K/Akt/mTOR pathway in breast cancer available on international databases—The Cancer Genome Atlas—and compared them with our own research. The analysis of the data contained in TCGA confirmed the lower level of *PIK3CA*, *PIK3R1* and *PTEN* gene expression in breast cancer tissues compared to normal tissues and a higher level of *AKT1* gene. Additionally confirmed was a statistically significant difference between the expression of the *AKT1*, *PTEN* genes and HER2 status. Moreover, the correlation analysis confirmed a statistically significant correlation between the majority of the studied genes. Differences between our results and those contained in the TCGA database may result from a relatively small group of patients participating in our study. Therefore, there is a need to confirm them in an independent cohort of patients. The publication by the TCGA program entitled “The Comprehensive molecular portraits of human breast tumours” analysed primary breast cancers among others in terms of mutations and mRNA-expression. The results of these studies indicate the importance of mutations in the *PIK3CA* gene in various types of breast cancer [55]. Studies conducted in recent years also point to the significance of the mutation of genes related to the PI3K/Akt/mTOR pathway and the level of expression of other genes, such as *CALM1*, *SLC4A8*, *NRK*, *CCNE1* [33,56].

## 4. Materials and Methods

### 4.1. Characteristics of the Studied Group

The studied material was obtained from tumor tissue perpetuated in paraffin blocks. The control population consisted of 11 female patients with mastopathy (age median ± SD:54 ± 13.53 range: 46–82). The cohort comprised 54 female patients with diagnosed ductal breast cancer (age median ± SD:58 ± 12.67, range: 37–92). In the analyzed population, the histological malignancy level of tumors, lymph node metastasis, ER, PR, HER2 status and Ki-67 index was determined. Specific characteristics of the studied group were presented in Table 5. The study was approved by the Bioethics Commission of the Medical University of Lublin–resolution no. KE-0254/135/2014.

### 4.2. Analysis of the Mutations of Selected Genes within the PI3K/Akt Pathway

DNA was extracted using the QIAamp DNA FFPE Tissue Kit (Qiagen, Hilden, Germany) according to the manufacturer’s instruction. 

**Figure 8:**
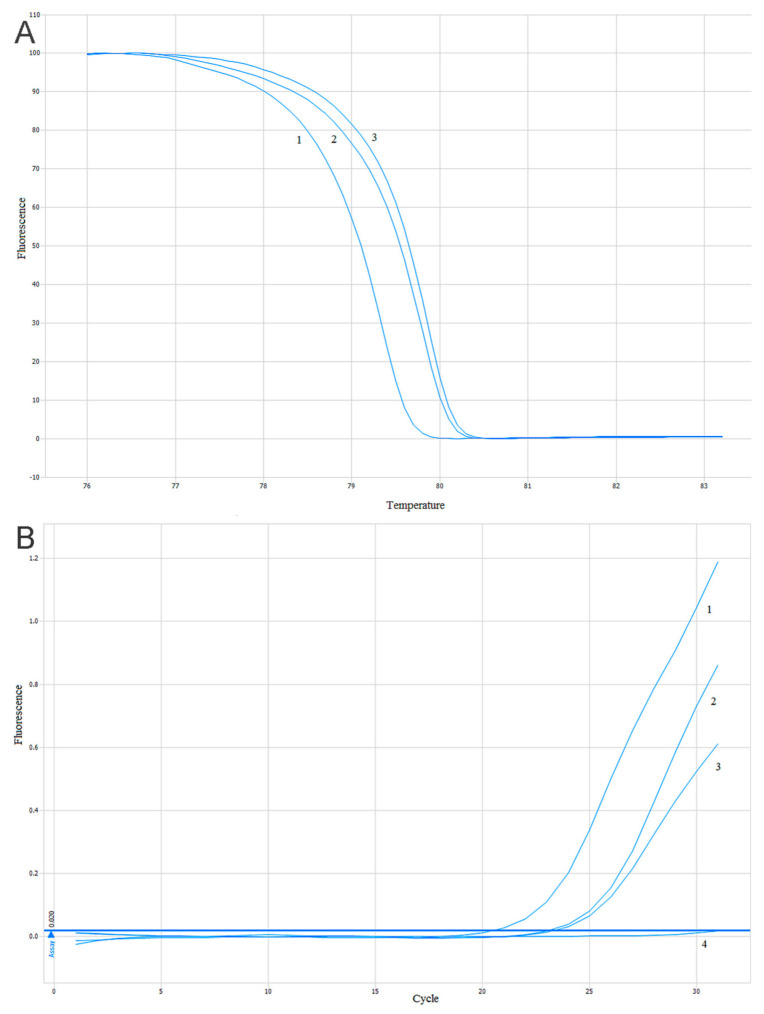
Sample analysis of the results of the High-Resolution Melt PCR (HRM-PCR) and Allele-Specific-quantitative PCR ASP-qPCR reactions: (**A**) analysis of the melting curves of the HRM-PCR reaction products for the *PIK3CA* gene. 1. Negative control. 2. Studied sample. 3. Positive control; (**B**) analysis of the ASP-qPCR reaction product amplification for the *PIK3CA* gene. 1. Negative control. 2. Studied sample. 3. Positive control. 4. Negative DNA control [57].

The estimation of the *PIK3CA* gene mutations (substitutions E542K, E545K, H1047R) was conducted using two methods based on a quantitative real-time PCR (qPCR). The High-Resolution Melt PCR (HRM-PCR) technique was used as a screening method with DNA intercalating dye and two pairs of primers flanking the mutated site of the *PIK3CA* gene. One pair of primers was flanking the mutations located in exon 9 (substitutions E542K and E545K) and the second pair of primers was flanking a mutation located in exon 20 (substitution H1047R). The reaction was performed according to the protocol described previously [57]. A comparison of the amplification and melting curves in the positive and negative controls allowed us to distinguish the mutant (mt) and wild-type (wt) samples–Figure 8A. To confirm the results of the HRM-PCR screening tests and identifications of diagnosed mutations in the *PIK3CA* gene, a reaction of the allele-specific and quality PCR was carried out. In addition, the design of the technique and result analysis was previously described [57] and shown in Figure 8B. 

The positive control of the analysis was the HRM and ASP reactions with DNA from the PIK3CA gene mutation-positive cell-lines (SW48 cell-line: substitution E542K; MCF10A cell-line: substitution E545K and H1047R) that were provided by Horizon Discovery (Horizon Discovery, Cambridge, UK ). The negative control was determined with DNA isolated from peripheral blood leukocytes of healthy individuals. 

Mutation in the *AKT1* gene (E17K substitution) was assessed using hydrolyzing molecular probes TaqMan™ Mutation Detection Assays (Hs00000986_mu, Hs00001010_rf) (Applied Biosystems, Foster City, CA, USA ). The reaction was performed on 96-well reaction plates in a 7300 Real-Time PCR System device (Applied Biosystems, Foster City, CA, USA) in steps recommended by Applied Biosystems. The amplification protocol included the following steps 95 °C for 10 min and 92 °C for 15 s, 58 °C for 1 min. for five cycles, followed by 92 °C for 15 s, 60 °C for 60 s for 40 cycles [58].

### 4.3. Analysis of the Expression Level of Selected Genes within the PI3K/Akt/mTOR Pathway

RNA was extracted using the commercial MagMAX™-96 for Microarrays Total RNA Isolation Kit (Thermo Fisher Scientific, Waltham, MA, USA) in accordance with the manufacturer’s instruction and assessed using the NanoDrop ND-1000 spectrophotometer (Thermo Fisher Scientific, Waltham, MA, USA). 

To perform the analysis of the expression level of selected genes, the reverse transcription reaction was conducted to obtain cDNA on the mRNA matrix using the High Capacity cDNA Reverse Transcription Kit (Applied Biosystems, Foster City, CA, USA) reagents in accordance with the manufacturer’s instruction. 

To mark the expression level, a comparison of the gene expression level in the studied samples (*n* = 54) with the gene expression level in the control sample (calibrator, *n* = 11) was performed using the TaqMan Gene Expression Assays probe and starts with the complementary sequence to the sequence of two neighboring exons of the studied genes-*PIK3CA*–first Hs00907957_m1 (exon 20–21), second Hs00907965_m1 (exon 9–10), *PIK3R1*-Hs00933163_m1, *AKT1*-Hs00178289_m1, *PTEN*-Hs02621230_s1, *mTOR*-Hs00234522_m1, *GAPDH*-Hs99999905_m1. (Applied Biosystems, Foster City, CA, USA). 

The cDNA, was amplified by real-time gene expression analysis (qPCR) using the manufacturer’s SDS software (Applied Biosystems, Foster City, CA, USA). Triplicate qPCR reactions were conducted for each sample. A blind trial was always performed without a DNA target in order to exclude reagent contamination by foreign DNA. The reagents were mixed according to manufacturer protocol to reach 25 μL of reaction mixture, including 11.25 of cDNA. 

The reaction was performed in 96-well plates using a 7900HT Real-Time Fast System device (Applied Biosystems, Foster City, CA, USA) in the following stages: initial denaturation: 95 °C, 10 min, and 40 cycles, each composed of two temperatures: 95 °C, 15 s, and 60 °C, 1 min.). The number of PCR cycles after which the level of fluorescence exceeded the defined threshold cycle (CT) RQ Study Software (Applied Biosystems, Foster City, CA, USA) was used to calculate the number of examined DNA molecules present in the mixture at the onset of the reaction. The CT value for each sample of the endogenous control gene was used to normalize the level of the interesting gene expression. The relative level of gene expression was calculated according to the formula. Calculations of the CT differences between the target gene and the reference gene are presented below: for the examined breast cancer: ΔCT breast cancer sample = CT target gene from breast cancer sample—CT reference gene, breast cancer sample and for mastopathy (control sample): ΔCT calibrator = CT target gene from control sample CT reference gene, control sample. Normalizing the ΔCT of the breast cancer sample to the ΔCT of the calibrator ΔΔCT = ΔCT breast cancer sample–ΔCT calibrator. The relative expression (RQ) of patients’ genes was calculated by the following formula: RQ = 2–ΔΔCT. Finally, the RQs were analyzed after their logarithmic conversion into the logarithm of RQ (LogRQ). Thus, the obtained results were legible as follows: LogRQ = 0: no difference between gene expression in the calibrated samples and the studied group. LogRQ < 0: decreased gene expression in the studied group, whereas LogRQ > 0: increased gene expression in the studied group compared to the calibrated sample [59,60,61].

### 4.4. Analysis of the Data Present on the PI3K/Akt/mTOR Pathway in Breast Cancer Available on International Database TCGA

For in-depth analysis of the data contained in The Cancer Genome Atlas (TCGA) data, used publicly available online sources Ualcan—http://ualcan.path.uab.edu/ (accessed on 19 February 2021) and The Breast Cancer Gene-Expression Miner v4.5 (bc-GenExMiner v4.5, http://bcgenex.centregauducheau.fr/BC-GEM (accessed on 19 February 2021)) [62,63,64]. 

### 4.5. Statistical Analysis

U Mann–Whitney test, χ2 Pearson test, Kruskal–Wallis test and r-Spearman correlation coefficient were performed using STATISTICA vs. 17. The results were considered statistically significant at *p* < 0.05. Heatmaps of gene expression were made using the Heatmapper program [65].

## 5. Conclusions

In summary, the results of our study and data available on the international database TCGA indicated that the PI3K/Akt/mTOR pathway plays an important role in breast cancer. We demonstrated that in the group of female patients in whom mutations in the *PIK3CA* and *AKT1* genes were observed, the level of *PIK3CA* gene expression was statistically significantly lower than in the group of female patients without mutations. Moreover, the presence of mutations does depend on the Ki-67 index; however, it does not depend on the patients’ age, lymph node metastasis, ER, PR or HER2 status and histopathological malignancy level. According to our knowledge, we demonstrated, for the first time, that there is a very strong positive correlation between the levels of *AKT1* and *mTOR* gene expression in the case of patients with mutations and without mutations. There is a need to confirm our research in an independent cohort of patients. Currently, studies are being conducted on the presence of mutations and changes in the expression of the *PIK3CA*, *AKT* and *mTOR* genes in breast cancer; however, the results of these studies are still inconclusive and require further experiments. The presence of mutations and changes in the expression of genes related to the PI3K/Akt/mTOR pathway can constitute a useful diagnostic biomarker and may contribute to the development of new, effective and targeted breast cancer treatment methods.

## Figures and Tables

**Table 1:** Prevalence of mutations in all patients participating in the study.

Gene	Substitution	Incidence
*PIK3CA*	E542K	1 (1.85%)
	E545K	2 (3.7%)
	H1047R	10 (18.52%)
*AKT1*	E17K	4 (7.4%)

**Table 2:** Prevalence of mutations depending on clinical parameters.

Examined Feature	*p*
Age	0.704405
Bloom Richardson Scale	0.71222
ER Status	0.66227
PR Status	0.07722
HER2 Status	0.46270
Ki-67 Index	0.02008 *
Lymph Node Metastasis	0.84787

* *p* < 0.05.

**Table 3:** The expression level of studied genes depending on the presence of mutations.

Gene	*p*
*AKT1*	0.460034
*mTOR*	0.243305
*PIK3CA1*	0.006065 *
*PIK3CA2*	0.034344 *
*PIK3R1*	0.635797
*PTEN*	0.144665

* p < 0.05.

**Table 4:** The expression level of the studied genes in depending on clinical parameters in analyzed samples and TCGA samples (web source the bc-GenExMiner).

Clinical parameters	Gene	*p* Analyzed Samples	*p* TCGA Samples
Age	*AKT1*	0.9214	0.2375
	*mTOR*	0.6773	0.0502
	*PIK3CA*	(1) 0.8979	0.2942
		(2) 0.7078	
	*PIK3R1*	0.3250	0.2821
	*PTEN*	0.8625	0.2624
Bloom Richardson Scale	*AKT1*	0.0317 *	-
	*mTOR*	0.0154 *	-
	*PIK3CA*	(1) 0.3632	-
		(2) 0.6685	
	*PIK3R1*	0.7813	-
	*PTEN*	0.0202 *	-
ER Status	*AKT1*	0.6358	0.0004 *
	*mTOR*	0.8623	0.7480
	*PIK3CA*	(1) 0.7325	0.0285 *
		(2) 0.6308	
	*PIK3R1*	0.3250	0.0039 *
	*PTEN*	0.5345	<0.0001 *
PR Status	*AKT1*	0.5906	0.2708
	*mTOR*	0.5228	0.9331
	*PIK3CA*	(1) 0.1756	0.1313
		(2) 0.4190	
	*PIK3R1*	0.3447	<0.0001 *
	*PTEN*	0.6476	<0.0001 *
HER2 Status	*AKT1*	0.0377 *	0.0002 *
	*mTOR*	0.1052	0.2515
	*PIK3CA*	(1) 0.0855	0.0549
		(2) 0.4534	
	*PIK3R1*	0.2821	0.3693
	*PTEN*	0.0111 *	0.0227 *
Ki-67 Index	*AKT1*	0.0571	-
	*mTOR*	0.0939	-
	*PIK3CA*	(1) 0.1760	-
		(2) 0.6745	-
	*PIK3R1*	0.9928	-
	*PTEN*	0.1917	-
Node Status	*AKT1*	0.911346	0.4174
	*mTOR*	0.101923	0.2850
	*PIK3CA*	(1) 0.385262	0.7241
		(2) 0.959624	
	*PIK3R1*	0.911346	0.0228 *
	*PTEN*	0.640050	0.2651

*p < 0.05., *PIK3CA*: (1)—exon 20–21, (2)—exon 9–10

**Table 5:** Characteristics of the studied group.

Examined Feature	Variable	Value
**Control Group**		
Age		54 ± 13.53, 46–82 ^1^
Histopathological Diagnosis	*Laesio fibroso-cystica*	11 (100%)
ER Status	2+	6 (54.55%)
	3+	5 (45.45%)
PR Status	1+	8 (72.73%)
	2+	3 (27.27%)
HER2 Status	1+, without overexpression	11 (100%)
Ki-67 Index	5%	10 (90.91%)
	15%	1 (9.09%)
**Study Group**		
Age		58 ± 12.67, 37–92 ^1^
Histopathological Diagnosis	*Carcinoma ductale invasivum*	39 (72.22%)
	*Carcinoma ductale invasivum parti comedocarcinoma*	5 (9.26%)
	*Carcinoma partim ductale partim lobulare invasivum*	6 (11.11%)
	*Carcinoma ductale invasivum recidivans*	1 (1.85%)
	*Carcinoma ductale in situ*	3 (5.56%)
Grade of histopathological malignancy	G1	3 (5.56%)
	G2	41 (75.92%)
	G3	7 (12.96%)
	Labeled as diffuse	3 (5.56%)
Bloom Richardson Scale	Bloom I	3 (5.56%)
	Bloom II	41 (75.92%)
	Bloom III	7 (12.96%)
	Not specified	3 (5.56%)
ER Status	(−)	6 (11.11%)
	(1+)	2 (3.7%)
	(2+)	6 (11.11%)
	(3+)	36 (66.67%)
	Not marked	4 (7.41%)
PR Status	(−)	14 (25.93%)
	(1+)	2 (3.7%)
	(2+)	12 (22.22%)
	(3+)	22 (40.74%)
	Not marked	4 (7.41%)
HER2 Status	0, without overexpression	20 (37.04%)
	1+, without overexpression	18 (33.33%)
	2+, overexpression	2 (3.7%)
	3+, overexpression	9 (16.67%)
	Not marked	5 (9.26%)
Ki-67 Index	≤10%	19 (35.185%)
	>10%-≤50%	19 (35.185%)
	>50%-≤90%	11 (20.37%)
	Not marked	5 (9.26%)
Lymph Node Metastasis	Yes	11 (20.37%)
	No	43 (79.63%)

^1^ mean ± SD, range.

## Data Availability

The data that support the findings of this study are available from the corresponding author upon reasonable request.

## Supplementary Materials

Supplementary Materials can be found at https://www.mdpi.com/1422-0067/22/4/2061/s1
