# Supplementary material for: The Correlation of Mutations and Expressions of Genes within the PI3K/Akt/mTOR Pathway in Breast Cancer—A Preliminary Study
Source: Int J Mol Sci. 2021 Feb 19;22(4):2061. doi: 10.3390/ijms22042061 (PMC7922286; doi:10.3390/ijms22042061)
Supplement: Supplementary file 1 [file ijms-22-02061-s001.pdf]

International Journal of *Molecular Sciences*

MDPI

Supplementary Materials: The correlation of mutations and expressions of genes within the PI3K/Akt/mTOR pathway in breast cancer - a preliminary study.

Przemysław Kołodziej[1]*, Marcin Nicoś[2], Paweł A. Krawczyk[2], Jacek Bogucki[3], Agnieszka Karczmarczyk[4], Daniel Zalewski[1], Tomasz Kubrak[5], Elżbieta Kołodziej[6], Anna Makuch-Kocka[7],Barbara Madej-Czerwonka[8,9], Bartosz J. Płachno[10], Janusz Kocki[6] and Anna Bogucka-Kocka[1]*

[1]Chair and Department of Biology and Genetics, Medical University of Lublin, 20-093 Lublin, Poland; daniel.zalewski@umlub.pl

[2]Department of Pneumonology, Oncology and Allergology, Medical University of Lublin, 20-954 Lublin, Poland; marcin_nicos@interia.pl (MN); pawel.krawczyk@umlub.pl (PAK)

[3]Department of Organic Chemistry, Medical University of Lublin, 20-093 Lublin, Poland; jacek.bogucki@umlub.pl

[4]Department of Experimental Haematooncology, Medical University of Lublin, 20-093 Lublin, Poland; agnieszka.piechnik@umlub.pl

[5]Department of Biochemistry and General Chemistry, Faculty of Medicine, University of Rzeszow, 35-310 Rzeszów, Poland; kubrak.tomasz@gmail.com

[6]Department of Clinical Genetics, Medical University of Lublin, 20-080 Lublin, Poland; elzbieta.kolodziej@umlub.pl (EK); janusz.kocki@umlub.pl (JK)

[7]Department of Pharmacology, Medical University of Lublin, 20-059 Lublin, Poland; anna.makuch-kocka@umlub.pl

[8]Department of Breast Surgery, District Specialist Hospital of Stefan Cardinal Wyszynski in Lublin, 20-718 Lublin, Poland; barbara.madej-czerwonka@umlub.pl

[9]Department of Human Anatomy, Medical University of Lublin, 20-090 Lublin, Poland; barbara.madej-czerwonka@umlub.pl

[10]Department of Plant Cytology and Embryology, Institute of Botany, Faculty of Biology, Jagiellonian University in Kraków., 30-387 Kraków, Poland; ORCID: http://orcid.org/0000-0001-5579-5101, bartosz.plachno@uj.edu.p

*Correspondence: przemyslaw.kolodziej@umlub.pl (P.K.); annaboguckakocka@umlub.pl (A.B.-K), Tel.:+48814487236 (P.K.), Tel.: +48 814487232 (A.B.-K.)

Table S1. Prevalence of mutations depending on clinical parameters - the detailed statistical analysis.

Statistical analysis			
U Manna-Whitneya test			
Clinical parameters	U	Z	p
Age	284,0000	0,378868	0,704405
Ki-67 index	132,0000	2,309845	0,02008*
χ2 Pearson test			
Clinical parameters	χ2	df	p

Bloom Richardson Scale	1,371539	3	0,71222
ER status	1,587302	3	0,66227
PR status	6,838796	3	0,07722
HER2 status	2,570406	3	0,46270
Lymph node metastasis	0,0368032	1	0,84787

*p<0.05

Table S2. Differences between the expression level of the genes within the PI3K/Akt/mTOR pathway in normal tissues and breast cancer - analysis the data available on international database TCGA (web source the Ualcan).

**Comparison**	**Statistical significance**
AKT1	<1E-12
mTOR	5.967700E-02
PIK3CA	1.62447832963153E-12
PIK3R1	1.62447832963153E-12
PTEN	1.62447832963153E-12

Table S3. The expression level of studied genes depending on the presence of mutations - the detailed statistical analysis.

Statistical analysis			
U Manna-Whitneya test			
Gene	U	Z	p
AKT1	265,0000	0,738792	0,460034
mTOR	198,0000	1,166767	0,243305
PIK3CA1	121,0000	2,744285	0,006065*
PIK3CA2	104,0000	2,116015	0,034344*

International Journal of *Molecular Sciences* MDPI

PIK3R1	279,0000	0,473585	0,635797
PTEN	227,0000	1,458640	0,144665

*p<0.05

International Journal of *Molecular Sciences*

MDPI

Table S4. The expression level of studied *AKT1* gene depending on clinical parameters - the detailed statistical analysis.

Statistical analysis (LogRQ *AKT1*)					
Kruskala-Wallisa test					
Clinical parameters					
Age	H (2)=0,1636953 p =0,9214	1 R:28,094	2 R:27,333	3 R:25,800	
	1(37-58)		0,142792	0,402447	
	2(61-70)	0,142792		0,227629	
	3(72-92)	0,402447	0,227629		
Bloom Richardson Scale	H (3) =5,576581 p =0,1341	3 R:38,143	2 R:24,805	4 R:30,000	1 R:37,000
	3(Bloom III)		2,073112	0,750065	0,105272
	2(Bloom II)	2,073112		0,552121	1,296059
	4(Not specified)	0,750065	0,552121		0,544949
	1(Bloom I)	0,105272	1,296059	0,544949	
ER status	H (3)=1,705098 p =0,6358	0 R:32,667	1 R:24,000	2 R:25,833	3 R:24,333
	0 (-)		0,728146	0,811921	1,296407
	1(1+)	0,728146		0,154031	0,031476
	2(2+)	0,811921	0,154031		0,233353
	3(3+)	1,296407	0,031476	0,233353	
PR status	H (3) =1,913135 p =0,5906	0 R:30,071	1 R:23,500	2 R:23,667	3 R:23,773
	0 (-)		0,596347	1,116841	1,263850
	1(1+)	0,596347		0,014970	0,025332
	2(2+)	1,116841	0,014970		0,020274
	3(3+)	1,263850	0,025332	0,020274	
HER2 status	H (3) =8,445469 p =0,0377	0 R:27,850	1 R:17,722	2 R:25,000	3 R:33,222
	0 (-)		2,181630	0,268950	0,936696
	1(1+)	2,181630		0,683349	2,657143
	2(2+)	0,268950	0,683349		0,736100
	3(3+)	0,936696	2,657143	0,736100	
Ki-67 index	H(2)=6,113075 p =0,0571	1 R:22,632	2 R:31,053	3 R:18,636	
	1(≤10%)		1,816502	0,738007	
	2(>10% - ≤50%)	1,816502		2,293566	
	3(>50% - ≤90%)	0,738007	2,293566		

U Manna-Whitneya test					
Lymph node metastasis	U	Z	p		
	215,0000	-0,111340	0,911346		

International Journal of Molecular Sciences

MDPI

Table S5. The expression level of studied *mTOR* gene depending on clinical parameters - the detailed statistical analysis.

Statistical analysis (LogRQ *mTOR*)					
Kruskala-Wallisa test					
Clinical parameters					
Age	H(2)=0,7793797 p =0,6773	1 R:26,900	2 R:24,273	3 R:22,333	
	1(37-58)		0,511318	0,824270	
	2(61-70)	0,511318		0,295998	
	3(72-92)	0,824270	0,295998		
Bloom Richardson Scale	H(3)=9,409406 p =0,0243	3 R:35,857	2 R:21,757	4 R:35,667	1 R:37,333
	3(Bloom III)		2,346795	0,018935	0,146748
	2(Bloom II)	2,346795		1,589558	1,780017
	4(Not specified)	0,018935	1,589558		0,140028
	1(Bloom I)	0,146748	1,780017	0,140028	
ER status	H(3)=0,7460552 p =0,8623	0 R:28,000	1 R:27,000	2 R:24,400	3 R:22,657
	0 (-)		0,086026	0,399815	0,754168
	1(1+)	0,086026		0,231519	0,445027
	2(2+)	0,399815	0,231519		0,271590
	3(3+)	0,754168	0,445027	0,271590	
PR status	H(3)=2,246495 p =0,5228	0 R:26,846	1 R:11,500	2 R:23,667	3 R:23,600
	0 (-)		1,473539	0,579256	0,664538
	1(1+)	1,473539		1,161808	1,189940
	2(2+)	0,579256	1,161808		0,013316
	3(3+)	0,664538	1,189940	0,013316	
HER2 status	H(3)=6,135446 p =0,1052	0 R:27,389	1 R:17,444	2 R:24,500	3 R:28,125
	0 (-)		2,222617	0,288755	0,129063
	1(1+)	2,222617		0,705229	1,872627
	2(2+)	0,288755	0,705229		0,341610
	3(3+)	0,129063	1,872627	0,341610	
Ki-67 index	H(2)=4,731999 p =0,0939	1 R:20,389	2 R:29,118	3 R:19,909	
	1(≤10%)		0,163502	1,000000	
	2(>10% - ≤50%)	0,163502		0,228712	
	3(>50% - ≤90%)	1,000000	0,228712		

U Manna-Whitneya test					
Lymph node metastasis	U	Z	p		
	92,0000	1,635604	0,101923		

International Journal of Molecular Sciences

MDPI

Table S6. The expression level of studied *PIK3CA1* gene depending on clinical parameters - the detailed statistical analysis.

**Statistical analysis (LogRQ, *PIK3CA1*)**					
Kruskala-Wallisa test					
Clinical parameters					
Age	H(2)=0,2153703<br>p =0,8979	1<br>R:25,643	2<br>R:25,000	3<br>R:23,200	
	1(37-58)		0,126434	0,464080	
	2(61-70)	0,126434		0,288315	
	3(72-92)	0,464080	0,288315		
Bloom Richardson Scale	H(3)=4,605456<br>p =0,2031	3<br>R:29,000	2<br>R:22,842	4<br>R:38,000	1<br>R:32,667
	3(Bloom III)		0,905904	0,862483	0,351382
	2(Bloom II)	0,905904		1,768915	1,146519
	4(Not specified)	0,862483	1,768915		0,457143
	1(Bloom I)	0,351382	1,146519	0,457143	
ER status	H(3)=1,285727<br>p =0,7325	0<br>R:28,500	1<br>R:28,500	2<br>R:20,833	3<br>R:22,394
	0 (-)		0,000000	0,904311	0,878118
	1(1+)	0,000000		0,714920	0,638417
	2(2+)	0,904311	0,714920		0,267731
	3(3+)	0,878118	0,638417	0,267731	
PR status	H(3)=4,948413<br>p =0,1756	0<br>R:26,714	1<br>R:32,000	2<br>R:15,125	3<br>R:22,667
	0 (-)		0,532388	1,990947	0,893192
	1(1+)	0,532388		1,625209	0,960291
	2(2+)	1,990947	1,625209		1,382065
	3(3+)	0,893192	0,960291	1,382065	
HER2 status	H(3)=6,608244<br>p =0,0855	0<br>R:23,667	1<br>R:17,118	2<br>R:26,000	3<br>R:30,875
	0 (-)		1,507513	0,176805	1,320649
	1(1+)	1,507513		0,672008	2,498000
	2(2+)	0,176805	0,672008		0,357813
	3(3+)	1,320649	2,498000	0,357813	
Ki-67 index	H(2)=3,474769<br>p =0,1760	1<br>R:18,412	2<br>R:26,750	3<br>R:22,636	
	1(≤10%)		1,863630	0,849934	
	2(>10% - ≤50%)	1,863630		0,817633	
	3(>50% - ≤90%)	0,849934	0,817633		

U Manna-Whitneya test					
Lymph node metastasis	U	Z	p		
	160,0000	0,868243	0,385262		

International Journal of Molecular Sciences

Table S7. The expression level of studied *PIK3CA2* gene depending on clinical parameters - the detailed statistical analysis.

**Statistical analysis (LogRQ, *PIK3CA2*)**					
Kruskala-Wallisa test					
Clinical parameters					
Age	H(2)=,6912648 p =0,7078	1 R:20,000	2 R:23,091	3 R:23,222	
	1(37-58)		0,682290	0,663803	
	2(61-70)	0,682290		0,023815	
	3(72-92)	0,663803	0,023815		
Bloom Richardson Scale	H(3)=3,229627 p =0,3576	3 R:22,500	2 R:20,176	4 R:31,000	1 R:32,500
	3(Bloom III)		0,358309	0,800055	0,941242
	2(Bloom II)	0,358309		1,212562	1,380607
	4(Not specified)	0,800055	1,212562		0,122271
	1(Bloom I)	0,941242	1,380607	0,122271	
ER status	H(3)=1,727805 p =0,6308	0 R:29,000	1 R:20,500	2 R:19,400	3 R:19,833
	0 (-)		0,796486	1,124451	1,294924
	1(1+)	0,796486		0,112464	0,078087
	2(2+)	1,124451	0,112464		0,076737
	3(3+)	1,294924	0,078087	0,076737	
PR status	H(3)=2,827237 p =0,4190	0 R:14,500	1 R:22,500	2 R:18,111	3 R:21,947
	0 (-)		0,910577	0,668727	1,590044
	1(1+)	0,910577		0,505197	0,066894
	2(2+)	0,668727	0,505197		0,853087
	3(3+)	1,590044	0,066894	0,853087	
HER2 status	H(3)=2,623337 p =0,4534	0 R:20,500	1 R:16,353	2 R:27,500	3 R:20,750
	0 (-)		1,061563	0,855490	0,040738
	1(1+)	1,061563		1,377593	0,730979
	2(2+)	0,855490	1,377593		0,720064
	3(3+)	0,040738	0,730979	0,720064	
Ki-67 index	H(2)=0,7876709 p =0,6745	1 R:17,294	2 R:20,273	3 R:20,667	
	1(≤10%)		0,711137	0,755815	
	2(>10% - ≤50%)	0,711137		0,080971	
	3(>50% - ≤90%)	0,755815	0,080971		

U Manna-Whitneya test					
Lymph node metastasis	U	Z	p		
	121,0000	-0,050625	0,959624		

International Journal of *Molecular Sciences*

MDPI

Table S8. The expression level of studied *PIK3R1* gene depending on clinical parameters - the detailed statistical analysis.

**Statistical analysis (LogRQ, *PIK3R1*)**					
Kruskala-Wallisa test					
Clinical parameters					
Age	H(2)=0,2958923 p =0,8625	1 R:27,375	2 R:29,333	3 R:25,700	
	1(37-58)		0,367738	0,293885	
	2(61-70)	0,367738		0,539383	
	3(72-92)	0,293885	0,539383		
Bloom Richardson Scale	H(3)=8,832424 p =0,0316	3 R:23,857	2 R:25,585	4 R:49,667	1 R:40,000
	3(Bloom III)		0,268616	2,377399	1,486971
	2(Bloom II)	0,268616		2,559284	1,531942
	4(Not specified)	2,377399	2,559284		0,752549
	1(Bloom I)	1,486971	1,531942	0,752549	
ER status	H(3)=3,467582 p =0,3250	0 R:31,333	1 R:39,000	2 R:27,333	3 R:23,472
	0 (-)		0,644129	0,475271	1,222944
	1(1+)	0,644129		0,980196	1,466237
	2(2+)	0,475271	0,980196		0,600669
	3(3+)	1,222944	1,466237	0,600669	
PR status	H(3)=3,321477 p =0,3447	0 R:26,929	1 R:21,000	2 R:19,333	3 R:28,364
	0 (-)		0,538009	1,324433	0,287949
	1(1+)	0,538009		0,149696	0,683965
	2(2+)	1,324433	0,149696		1,726177
	3(3+)	0,287949	0,683965	1,726177	
HER2 status	H(3)=3,815265 p =0,2821	0 R:27,150	1 R:19,944	2 R:32,000	3 R:28,778
	0 (-)		1,552153	0,457686	0,283818
	1(1+)	1,552153		1,131960	1,514286
	2(2+)	0,457686	1,131960		0,288472
	3(3+)	0,283818	1,514286	0,288472	
Ki-67 index	H(2)=0,0144830 p =0,9928	1 R:24,842	2 R:24,895	3 R:25,455	
	1(≤10%)		0,011353	0,113132	
	2(>10% - ≤50%)	0,011353		0,103409	
	3(>50% - ≤90%)	0,113132	0,103409		

U Manna-Whitneya test					
Lymph node metastasis	U	Z	p		
	215,0000	-0,111340	0,911346		

International Journal of Molecular Sciences

Table S9. The expression level of studied *PTEN* gene depending on clinical parameters - the detailed statistical analysis.

**Statistical analysis (LogRQ, *PTEN*)**					
Kruskala-Wallisa test					
Clinical parameters					
Age	H(2)=0,2168939 p =0,8972	1 R:27,781	2 R:25,750	3 R:28,700	
	1(37-58)		0,381430	0,161198	
	2(61-70)	0,381430		0,437939	
	3(72-92)	0,161198	0,437939		
Bloom Richardson Scale	H(3)=8,435348 p =0,0378	3 R:39,857	2 R:24,244	4 R:41,333	1 R:29,333
	3(Bloom III)		2,426754	0,135977	0,969382
	2(Bloom II)	2,426754		1,816211	0,540889
	4(Not specified)	0,135977	1,816211		0,934199
	1(Bloom I)	0,969382	0,540889	0,934199	
ER status	H(3)=2,187190 p =0,5345	0 R:33,500	1 R:22,000	2 R:23,000	3 R:24,778
	0 (-)		0,966193	1,247586	1,356906
	1(1+)	0,966193		0,084017	0,262296
	2(2+)	1,247586	0,084017		0,276567
	3(3+)	1,356906	0,262296	0,276567	
PR status	H(3)=1,652065 p =0,6476	0 R:29,571	1 R:20,500	2 R:24,583	3 R:23,864
	0 (-)		0,823219	0,869808	1,145283
	1(1+)	0,823219		0,366756	0,312428
	2(2+)	0,869808	0,366756		0,137573
	3(3+)	1,145283	0,312428	0,137573	
HER2 status	H(3)=11,11195 p =0,0111*	0 R:27,300	1 R:16,778	2 R:34,000	3 R:34,333
	0 (-)		0,140489	1,000000	1,000000
	1(1+)	0,140489		0,635159	0,015699
	2(2+)	1,000000	0,635159		1,000000
	3(3+)	1,000000	0,015699	1,000000	
Ki-67 index	H(2)=3,303943 p =0,1917	1 R:21,421	2 R:29,579	3 R:23,273	
	1(≤10%)		1,759736	0,342046	
	2(>10% - ≤50%)	1,759736		1,164902	
	3(>50% - ≤90%)	0,342046	1,164902		
U Manna-Whitneya test					
Lymph node metastasis	U	Z	p		
	199,0000	0,467630	0,640050		

International Journal of
Molecular Sciences

MDPI

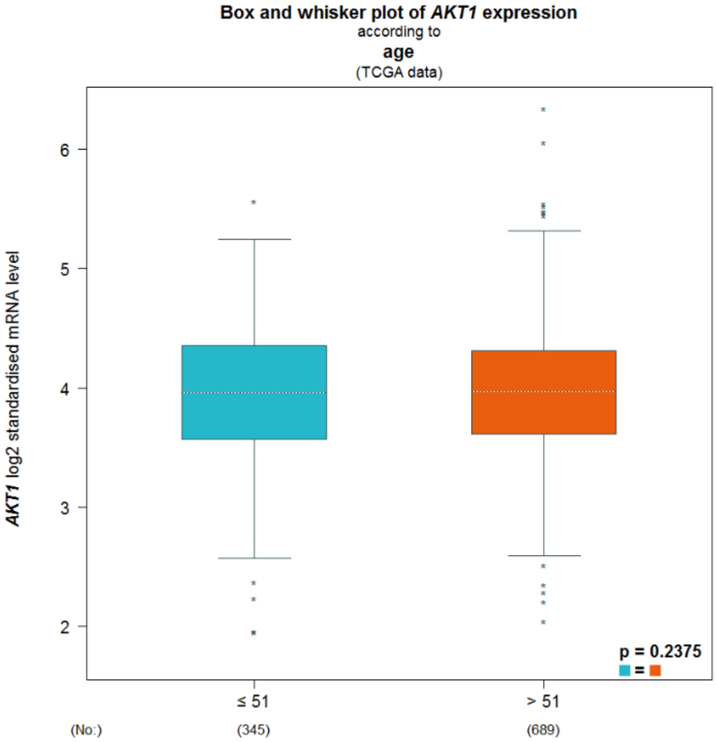

(Figure S1, A)

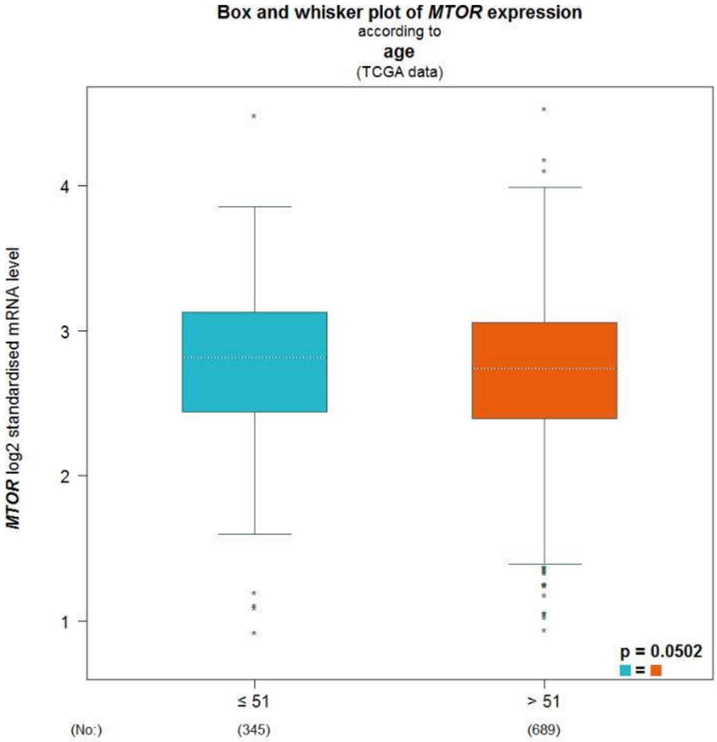

(Figure S1, B)

International Journal of
Molecular Sciences

MDPI

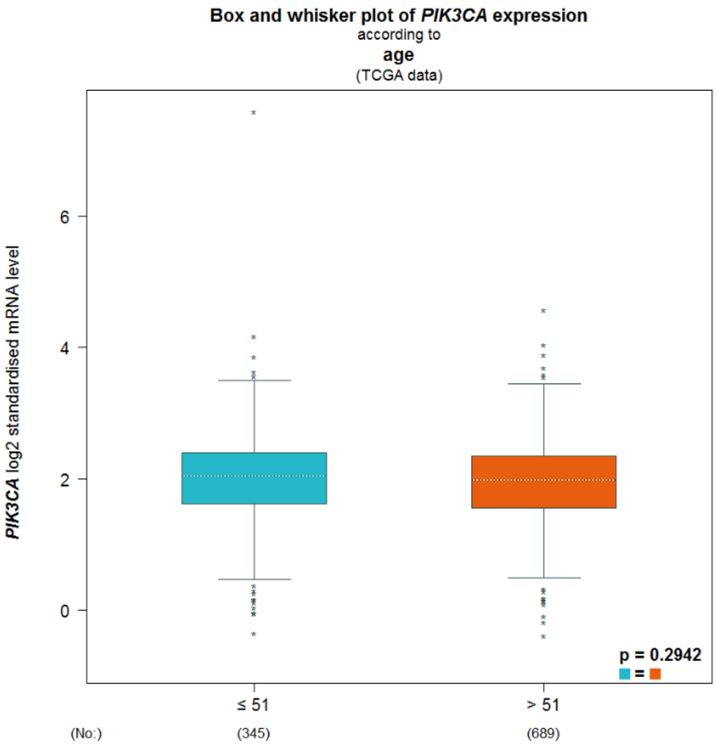

(Figure S1 C)

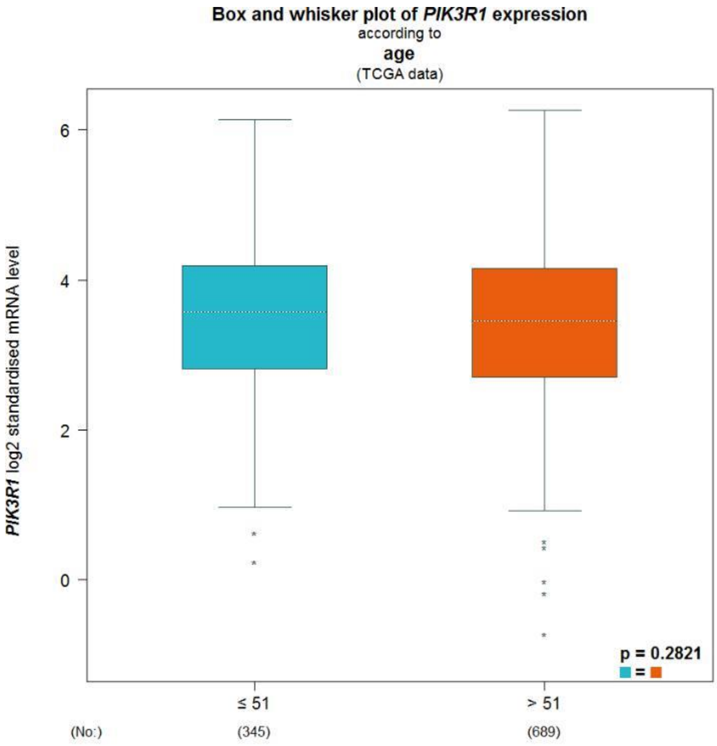

(Figure S1, D)

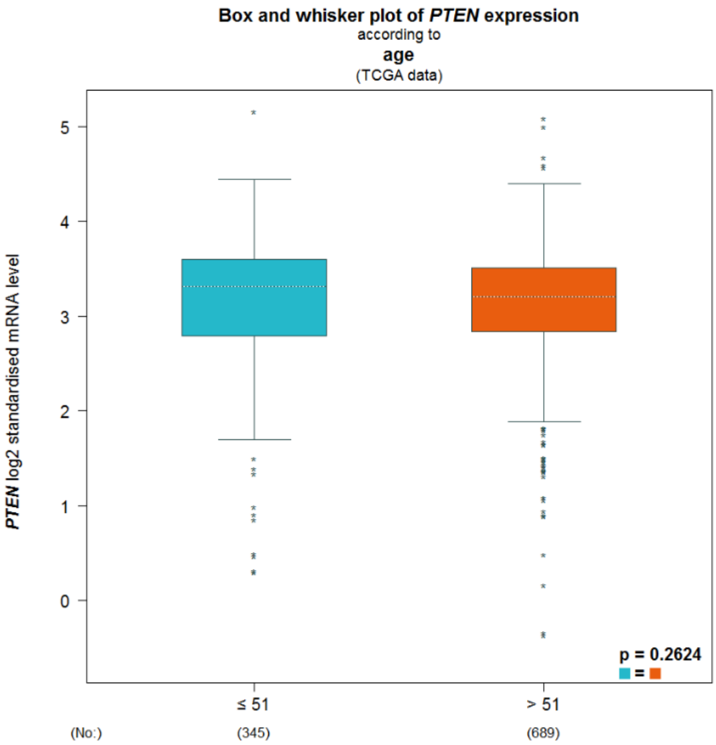

(Figure S1, E)

Figure S1. Comparison of the expression level of the genes within the PI3K/Akt/mTOR pathway in breast cancer samples from TCGA database depending on the age: *AKT1* (A), *MTOR* (B), *PIK3CA* (C), *PIK3R1* (D), *PTEN* (E) (web source the bc-GenExMiner).

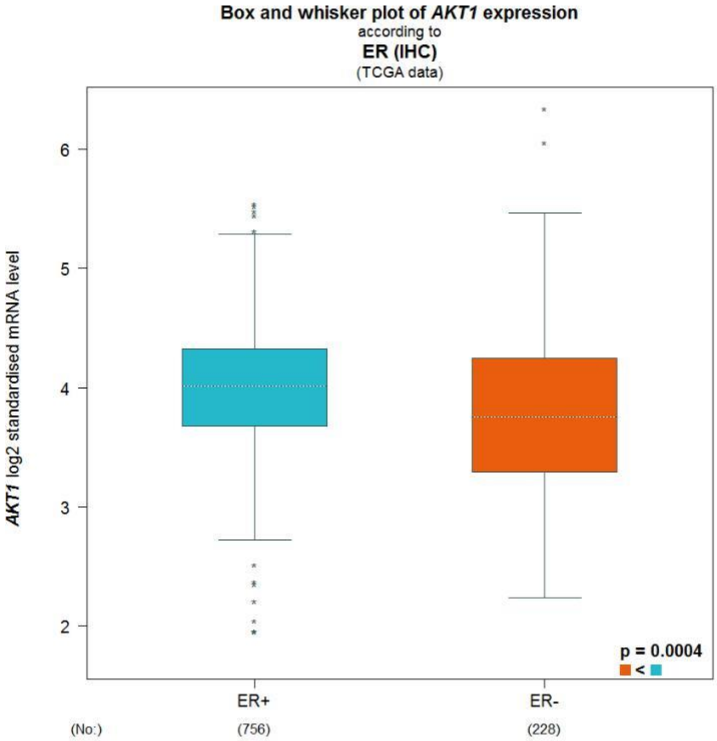

(Figure S2, A)

International Journal of Molecular Sciences

MDPI

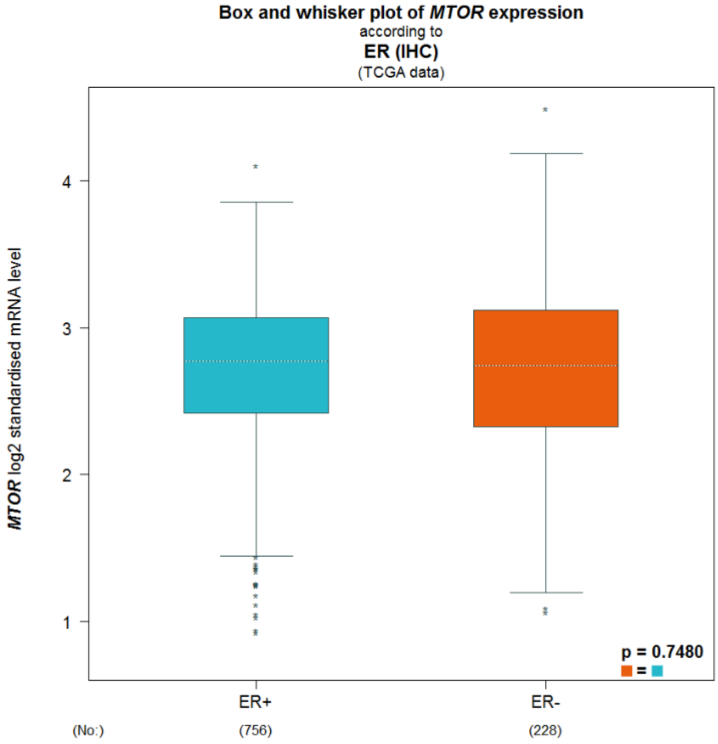

(Figure S2, B)

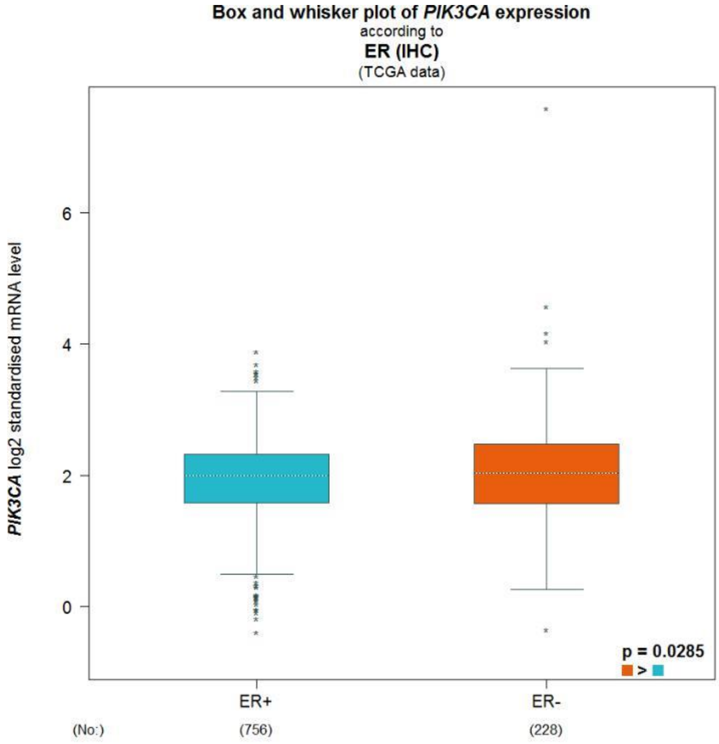

(Figure S2, C)

International Journal of
Molecular Sciences

MDPI

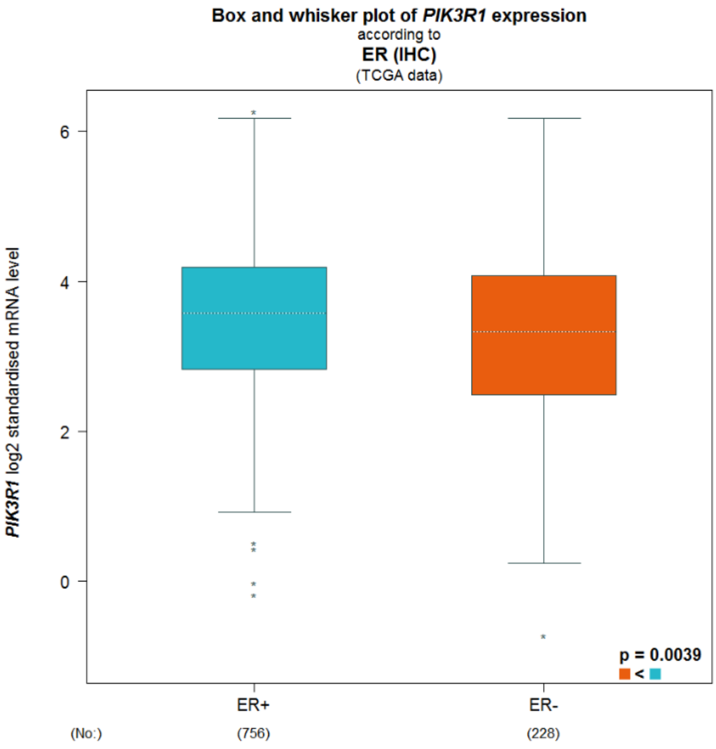

(Figure S2, D)

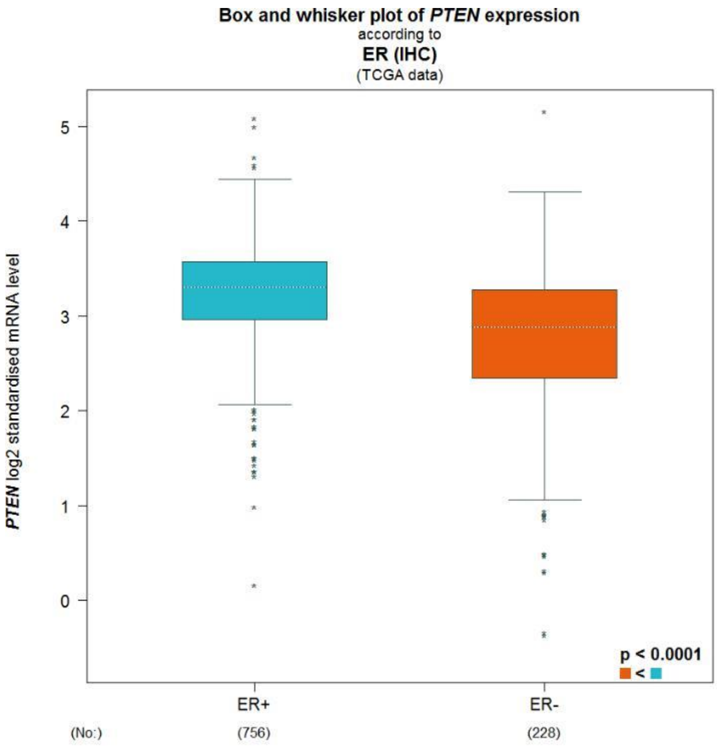

(Figure S2, E)

Figure S2. Comparison of the expression level of the genes within the PI3K/Akt/mTOR pathway in breast cancer samples from TCGA database depending on the ER status: *AKT1* (A), *MTOR* (B), *PIK3CA* (C), *PIK3R1* (D), *PTEN* (E) (web source the bc-GenExMiner).

International Journal of Molecular Sciences

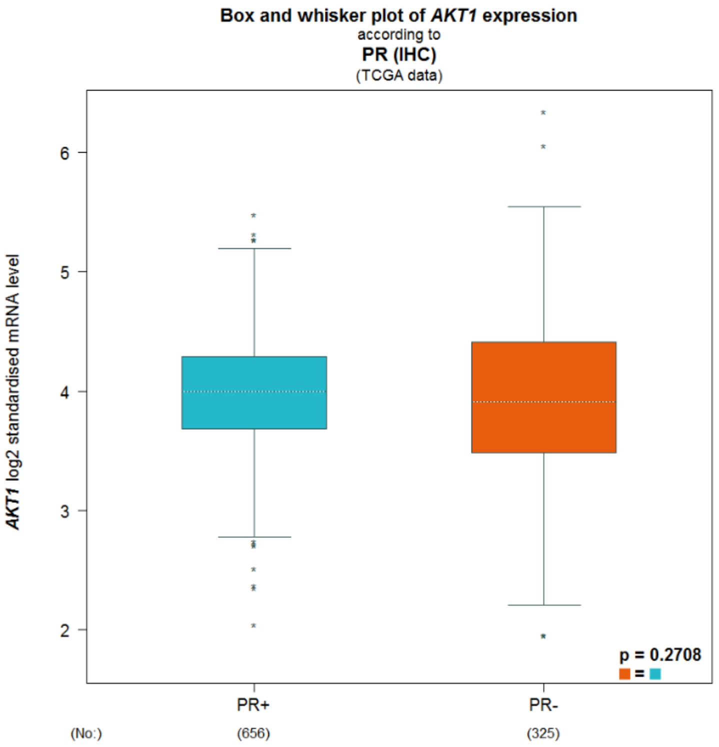

(Figure S3, A)

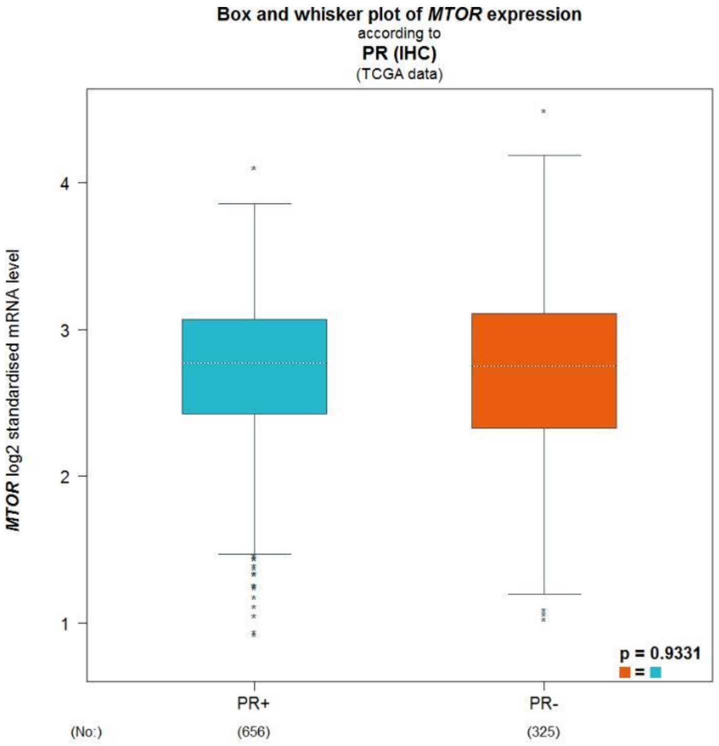

(Figure S3, B)

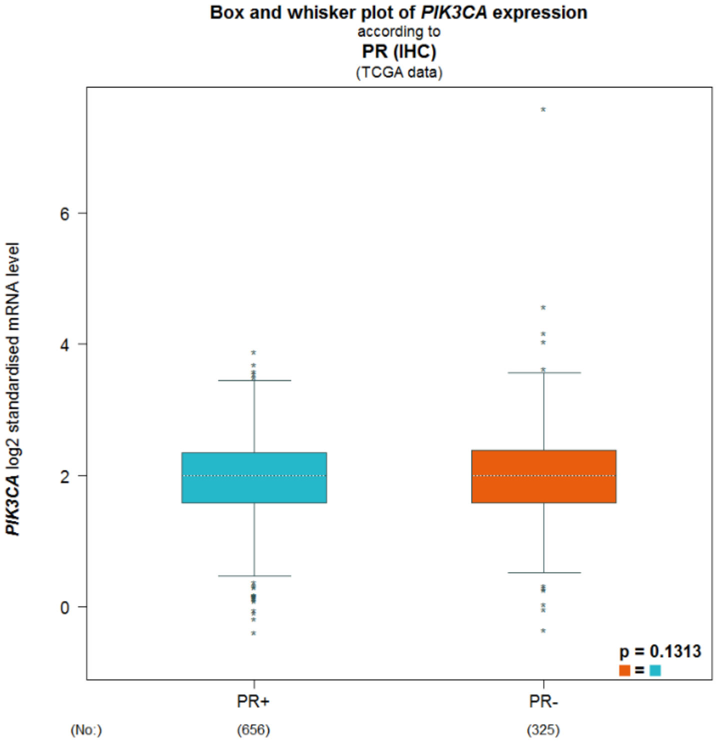

(Figure S3, C)

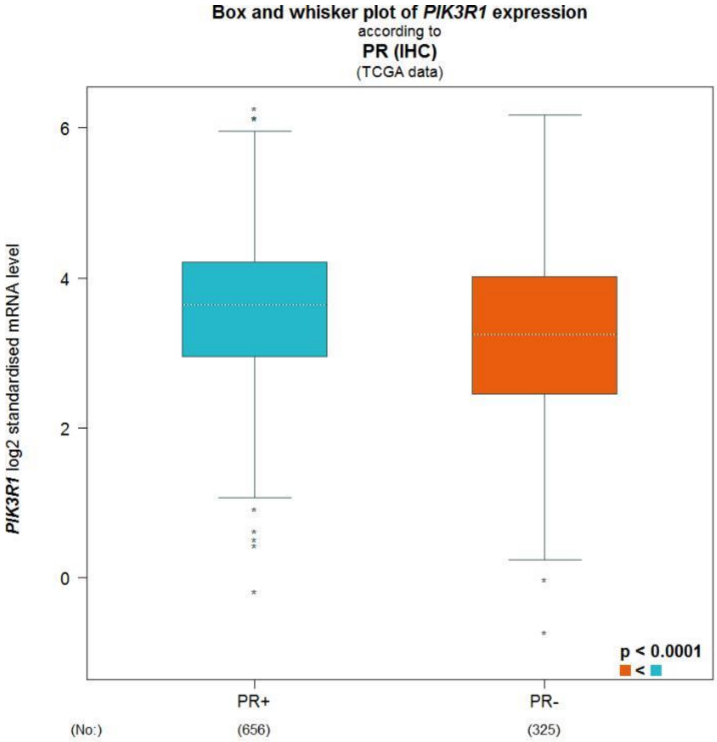

(Figure S3, D)

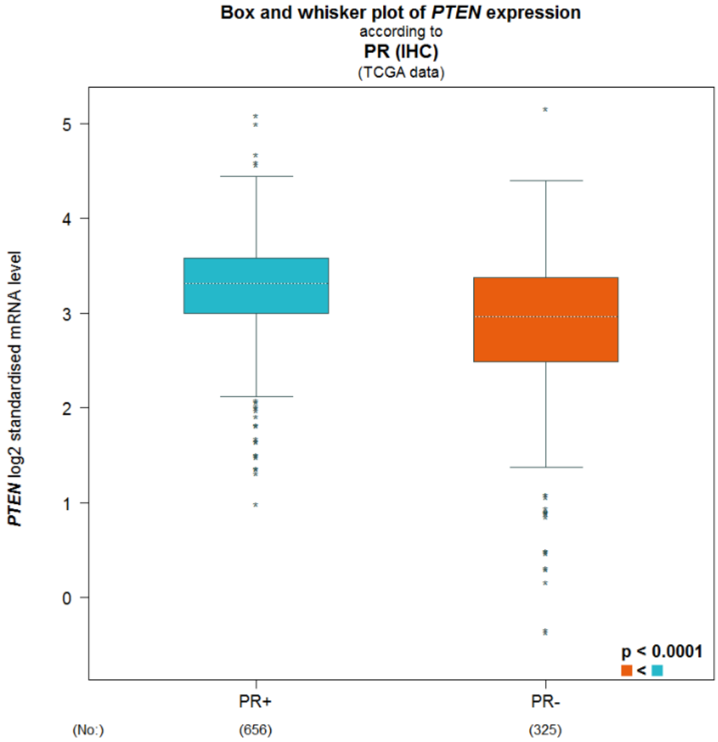

(Figure S3, E)

Figure S3. Comparison of the expression level of the genes within the PI3K/Akt/mTOR pathway in breast cancer samples from TCGA database depending on the PR status: *AKT1* (A), *MTOR* (B), *PIK3CA* (C), *PIK3R1* (D), *PTEN* (E) (web source the bc-GenExMiner).

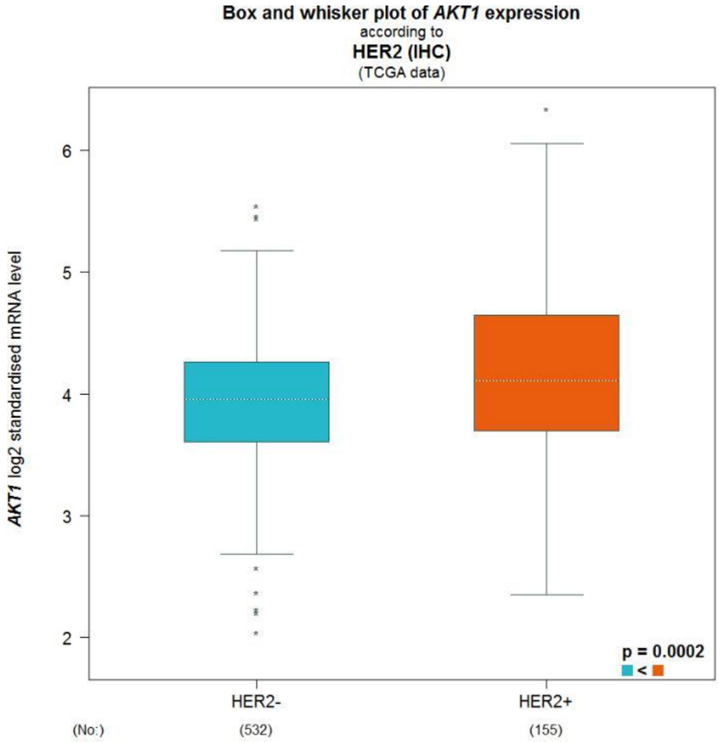

(Figure S4, A)

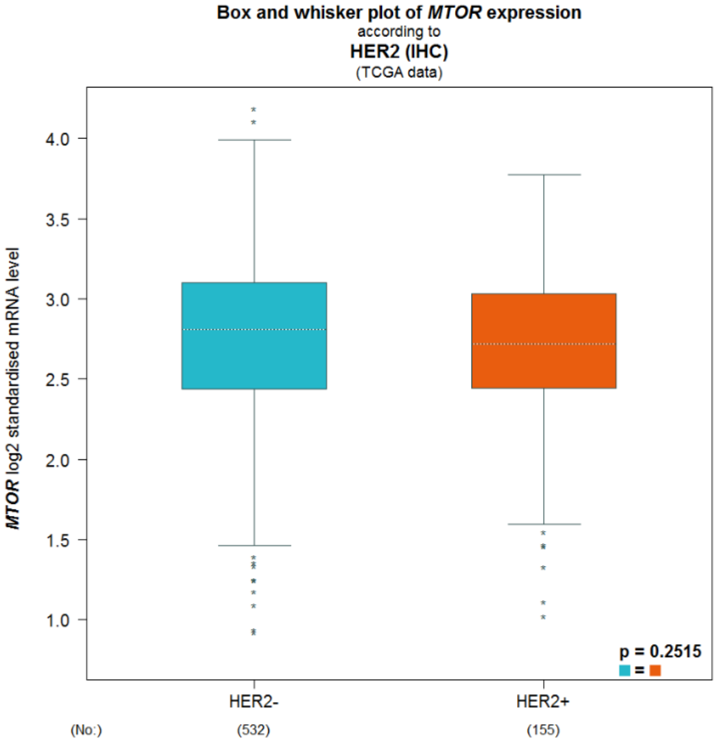

(Figure S4, B)

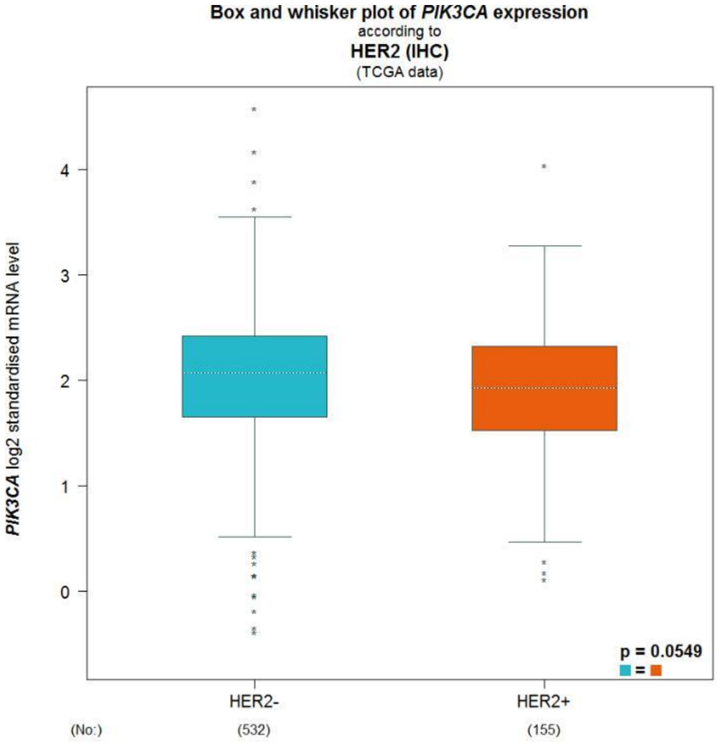

(Figure S4, C)

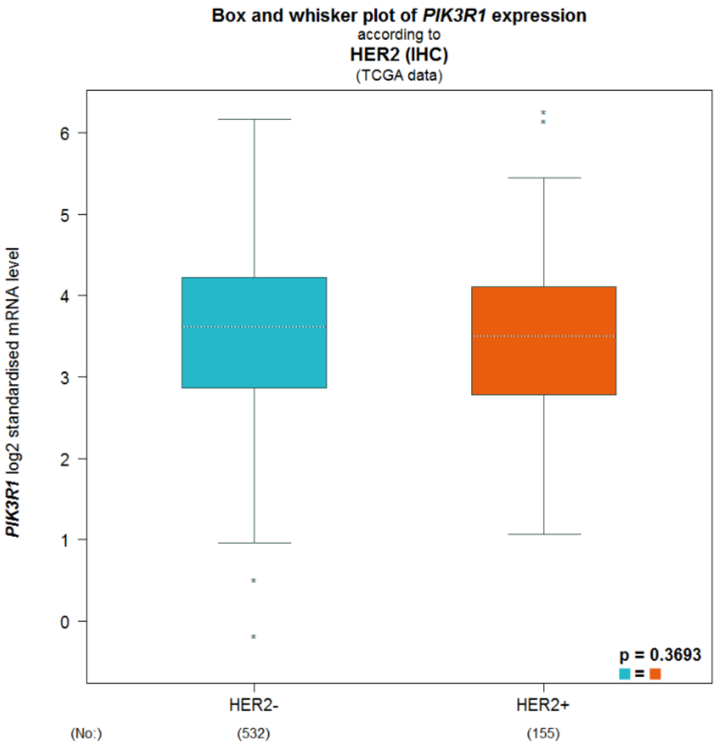

(Figure S4, D)

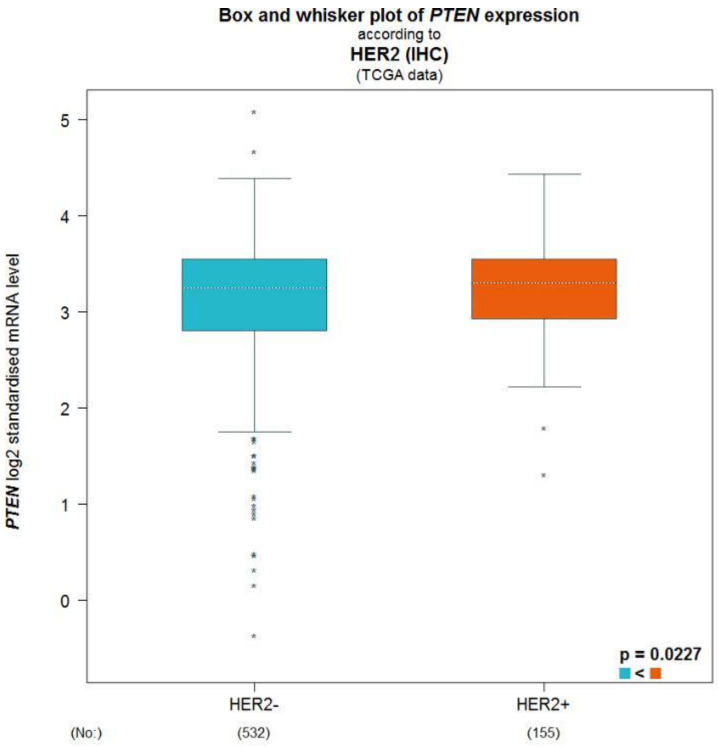

(Figure S4, E)

Figure S4. Comparison of the expression level of the genes within the PI3K/Akt/mTOR pathway in breast cancer samples from TCGA database depending on the HER2 status: *AKT1* (A), *MTOR* (B), *PIK3CA* (C), *PIK3R1* (D), *PTEN* (E) (web source the bc-GenExMiner).

International Journal of *Molecular Sciences*

MDPI

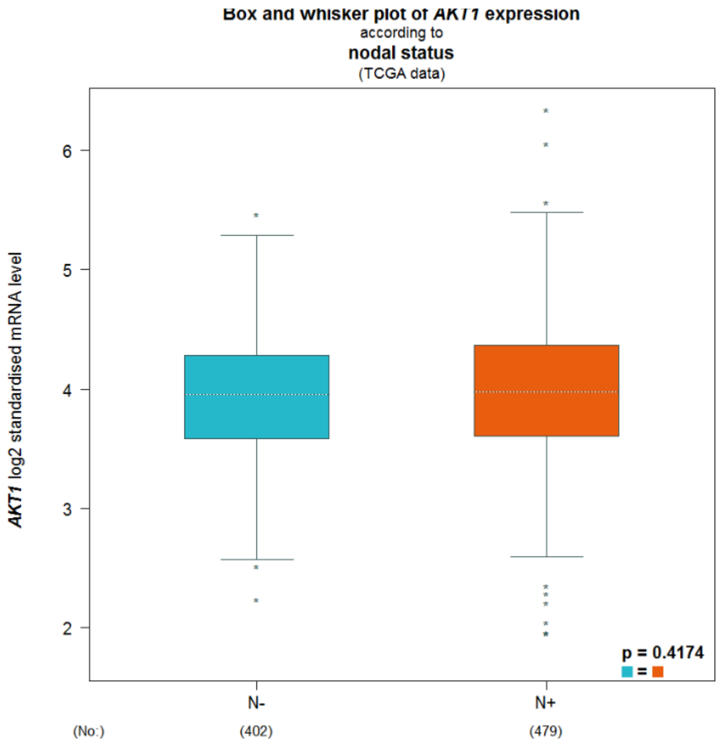

(Figure S5, A)

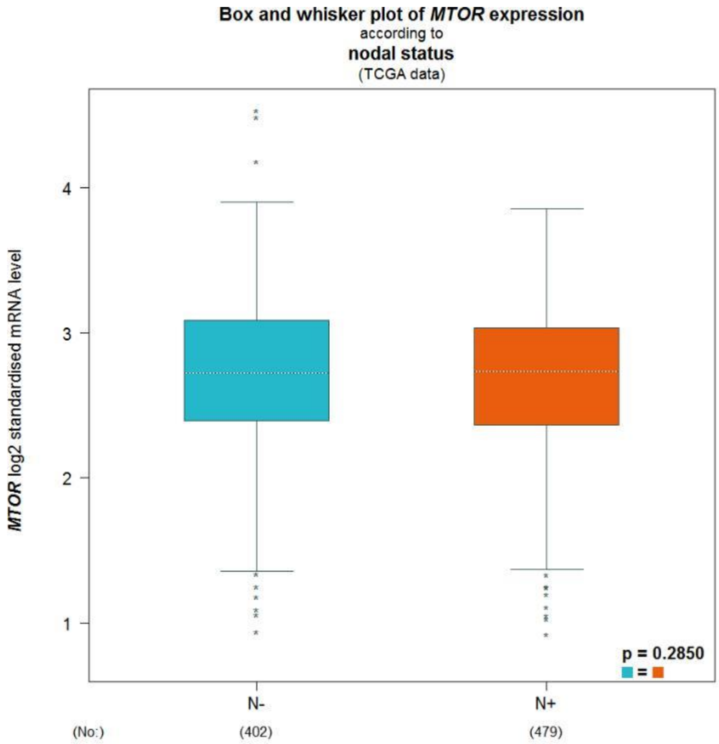

(Figure S5, B)

International Journal of
Molecular Sciences

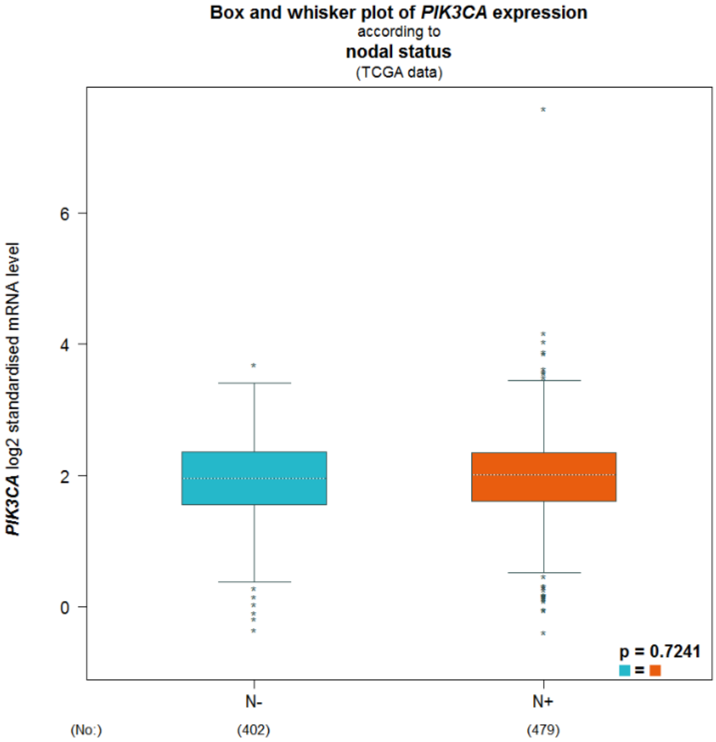

(Figure S5, C)

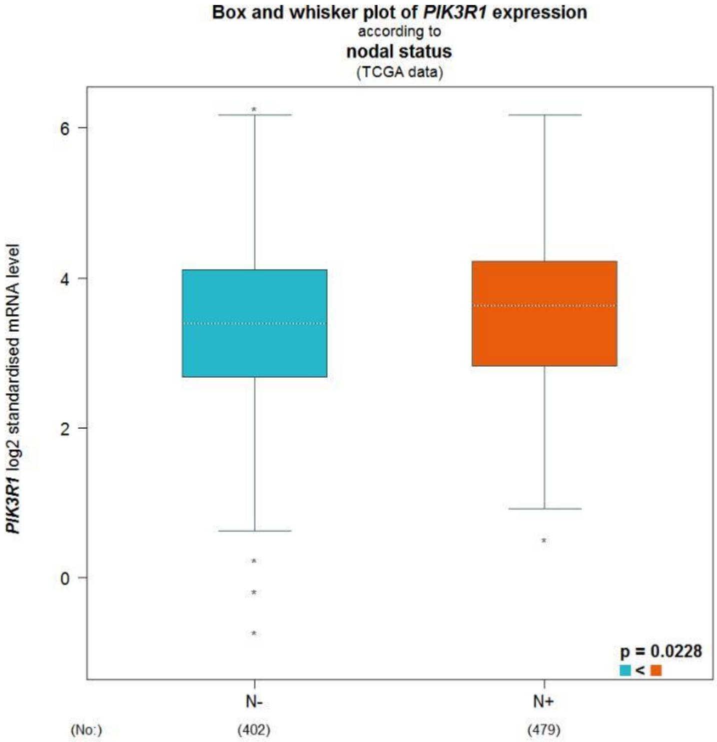

(Figure S5, D)

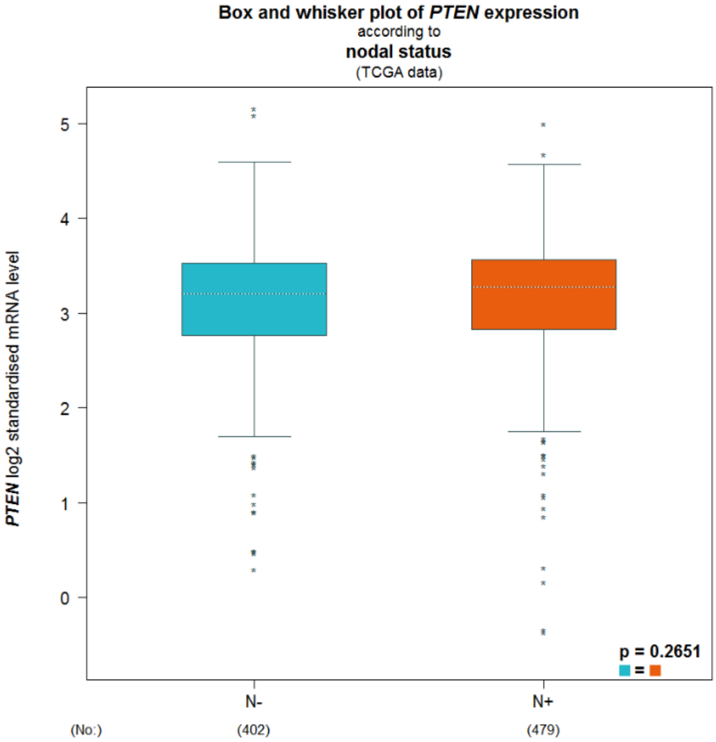

(Figure S5, E)

Figure S5. Comparison of the expression level of the genes within the PI3K/Akt/mTOR pathway in breast cancer samples from TCGA database depending on the nodal status: *AKT1* (A), *MTOR* (B), *PIK3CA* (C), *PIK3R1* (D), *PTEN* (E) (web source the bc-GenExMiner).

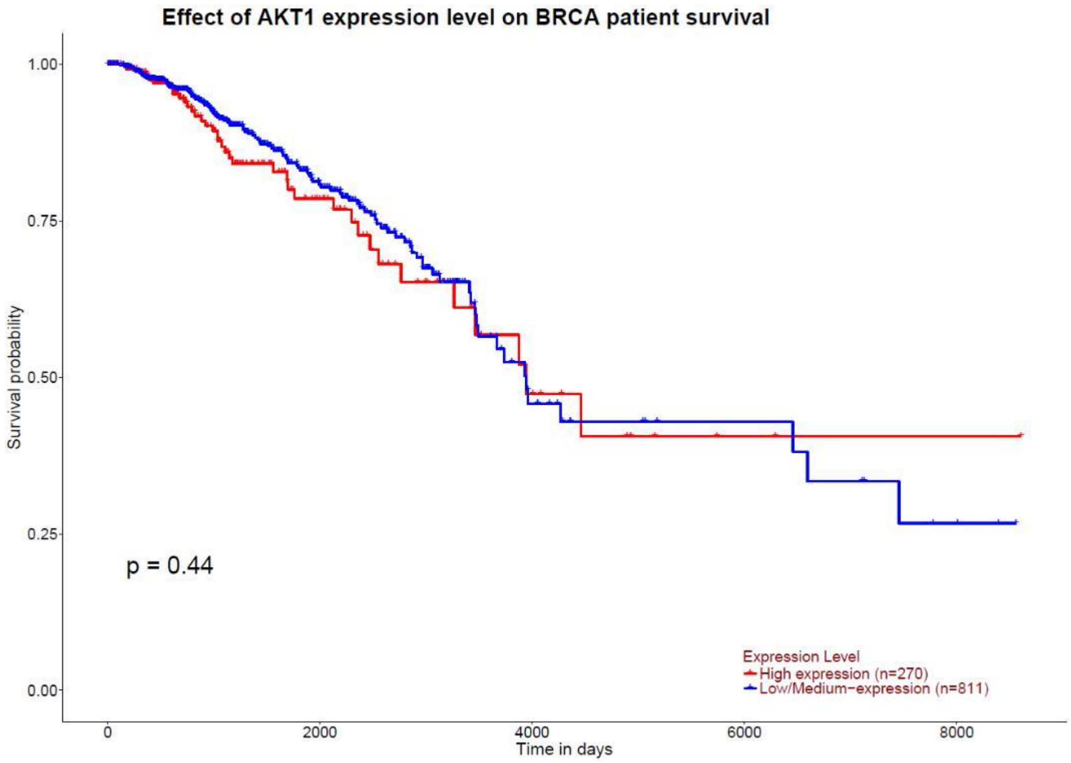

(Figure S6, A)

International Journal of
Molecular Sciences

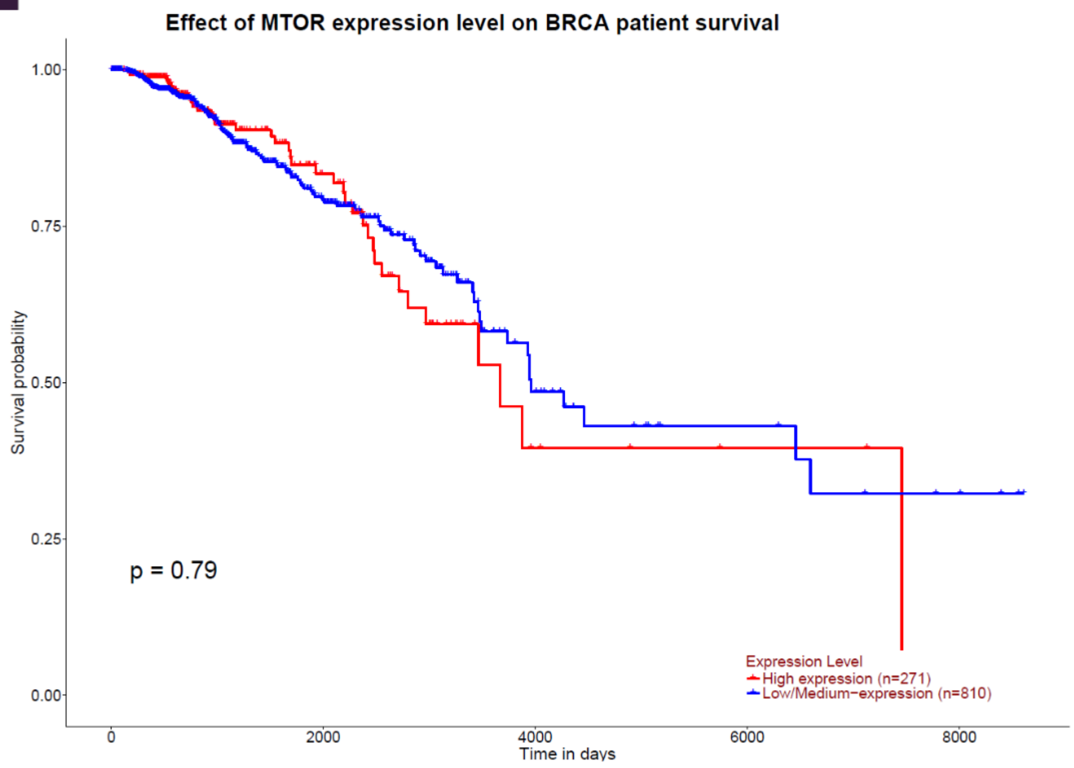

(Figure S6, B)

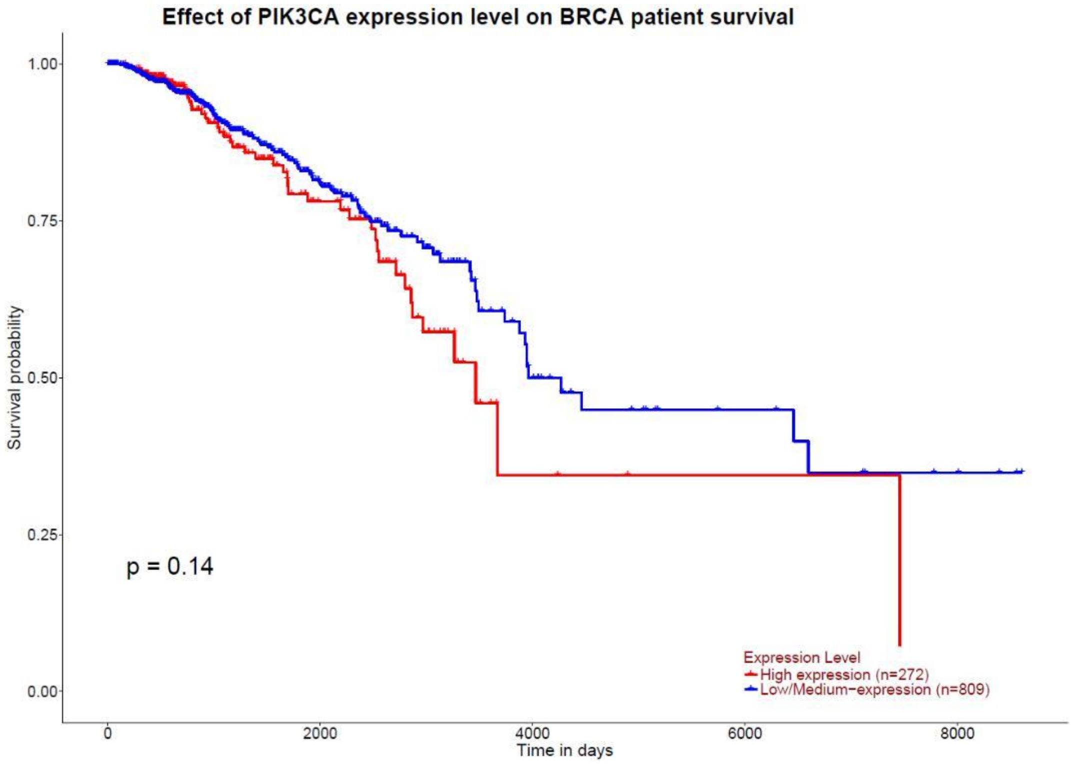

(Figure S6, C)

International Journal of
Molecular Sciences

MDPI

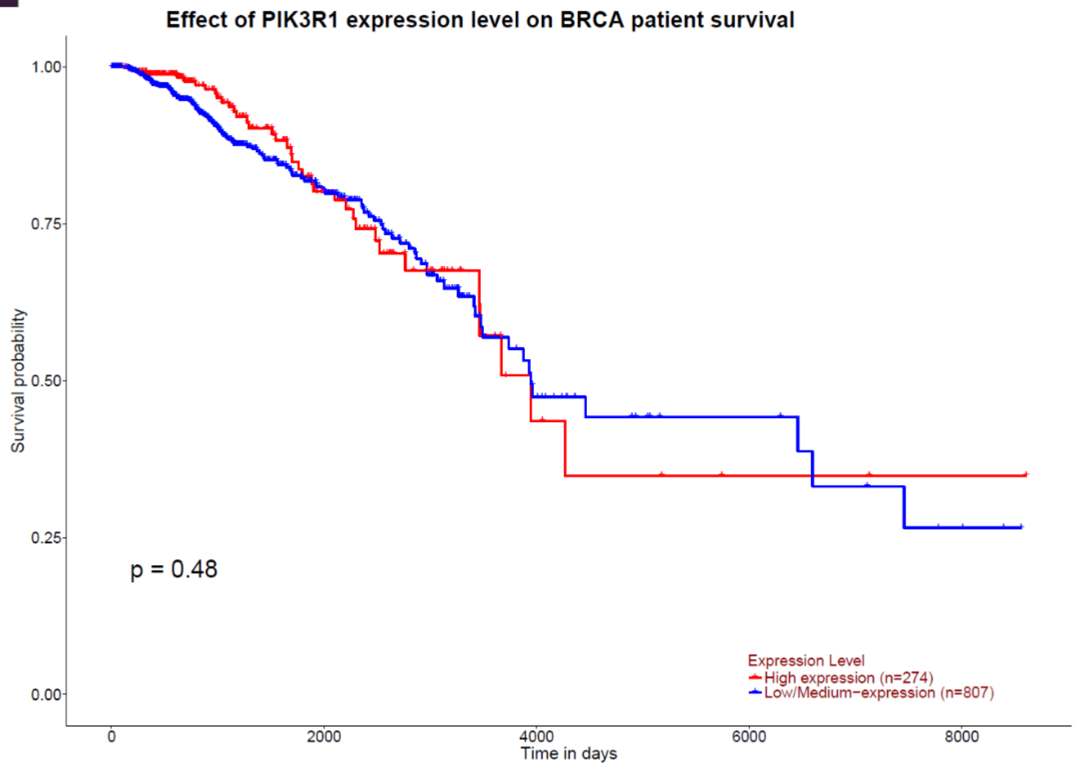

(Figure S6, D)

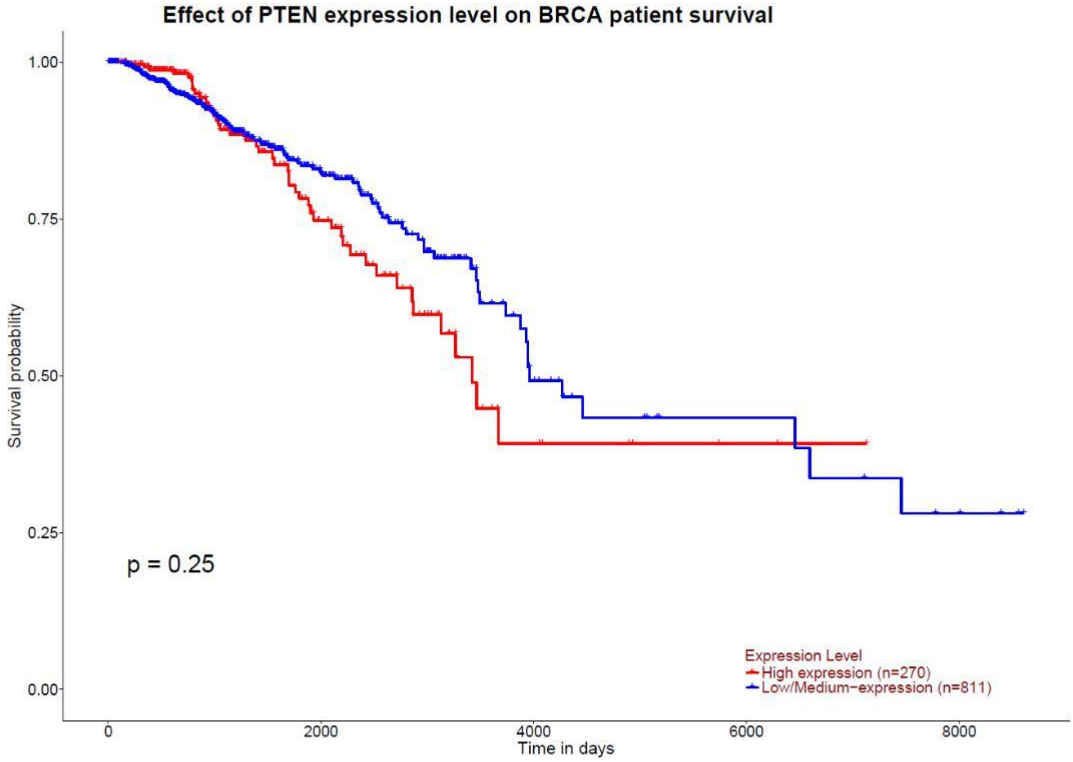

(Figure S6, E)

Figure S6. Effect of the expression level of the genes within the PI3K/Akt/mTOR pathway on breast cancer patients survival - analysis the data available on international database TCGA: *AKT1* (A), *MTOR* (B), *PIK3CA* (C), *PIK3R1* (D), *PTEN* (E) (web source the Ualcan).

International Journal of Molecular Sciences

Table S10. Correlation between the expression level of the studied genes using Pearson's correlation coefficient in brast cancer- analysis the data available on international database TCGA (web source the bc-GenExMiner). *p<0.05.

**Correlation table**	**Parameters**	***AKT1***	***MTOR***	***PIK3CA***	***PIK3R1***	***PTEN***
AKT1	Pearson's correlation coefficient	NA	0.12	-0.21	0.02	0.04
AKT1	p-value	NA	< 0.0001	< 0.0001	0.0968	0.0038
AKT1	No. patients	NA	4712	4712	4712	4712
MTOR	Pearson's correlation coefficient	0.12	NA	0.22	0.14	0.07
MTOR	p-value	< 0.0001	NA	< 0.0001	< 0.0001	< 0.0001
MTOR	No. patients	4712	NA	4712	4712	4712
PIK3CA	Pearson's correlation coefficient	-0.21	0.22	NA	0.23	0.22
PIK3CA	p-value	< 0.0001	< 0.0001	NA	< 0.0001	< 0.0001
PIK3CA	No. patients	4712	4712	NA	4712	4712
PIK3R1	Pearson's correlation coefficient	0.02	0.14	0.23	NA	0.31
PIK3R1	p-value	0.0968	< 0.0001	< 0.0001	NA	< 0.0001
PIK3R1	No. patients	4712	4712	4712	NA	4712
PTEN	Pearson's correlation coefficient	0.04	0.07	0.22	0.31	NA
PTEN	p-value	0.0038	< 0.0001	< 0.0001	< 0.0001	NA
PTEN	No. patients	4712	4712	4712	4712	NA

International Journal of
Molecular Sciences

MDPI

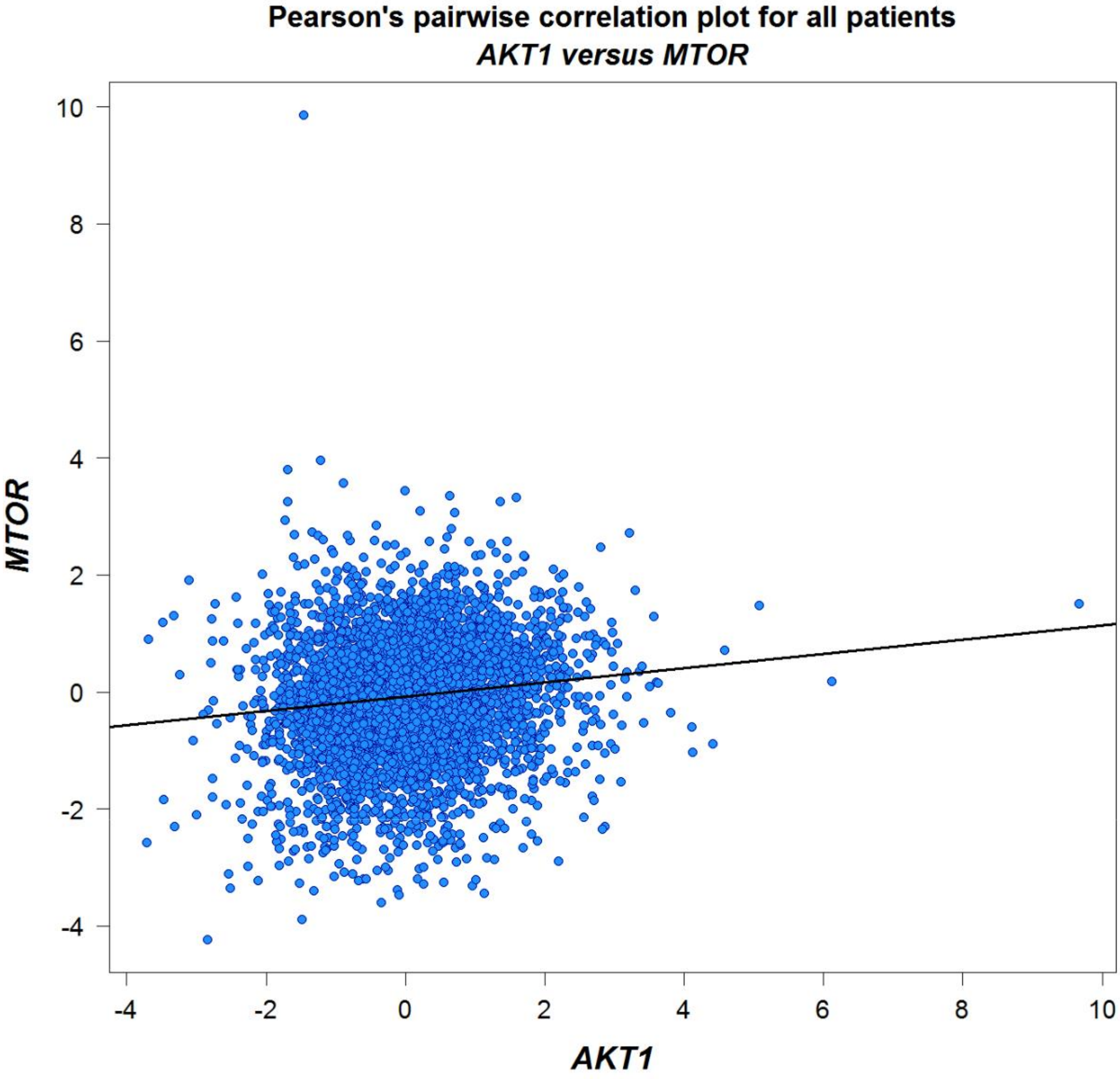

Figure S7. Correlation between the expression level of the *AKT1* and *MTOR* genes using Pearson's correlation coefficient in brast cancer- analysis the data available on international database TCGA (web source the bc-GenExMiner).

International Journal of
Molecular Sciences

MDPI

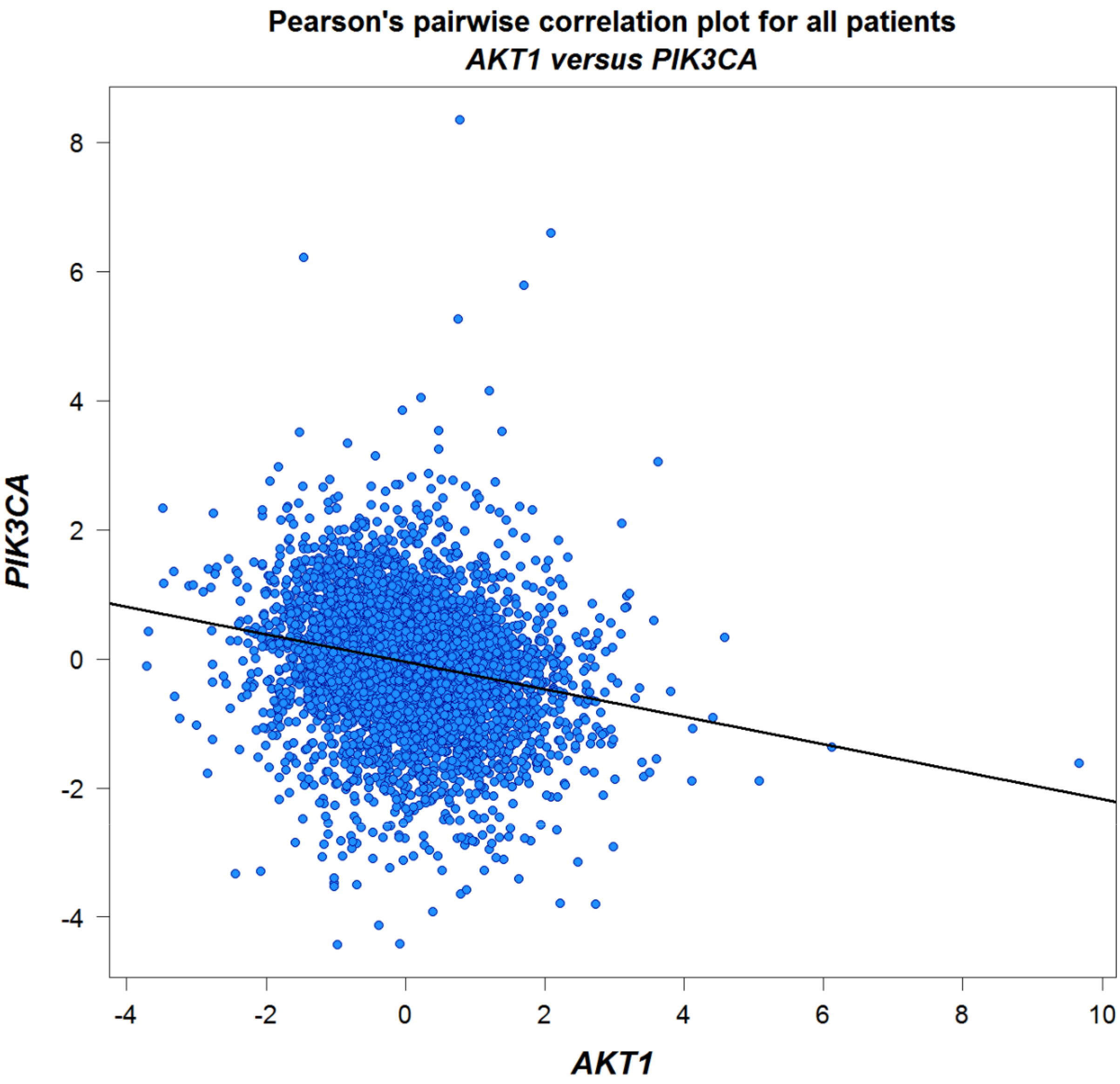

Figure S8. Correlation between the expression level of the *AKT1* and *PIK3CA* genes using Pearson's correlation coefficient in breast cancer- analysis the data available on international database TCGA (web source the bc-GenExMiner).

International Journal of *Molecular Sciences*

MDPI

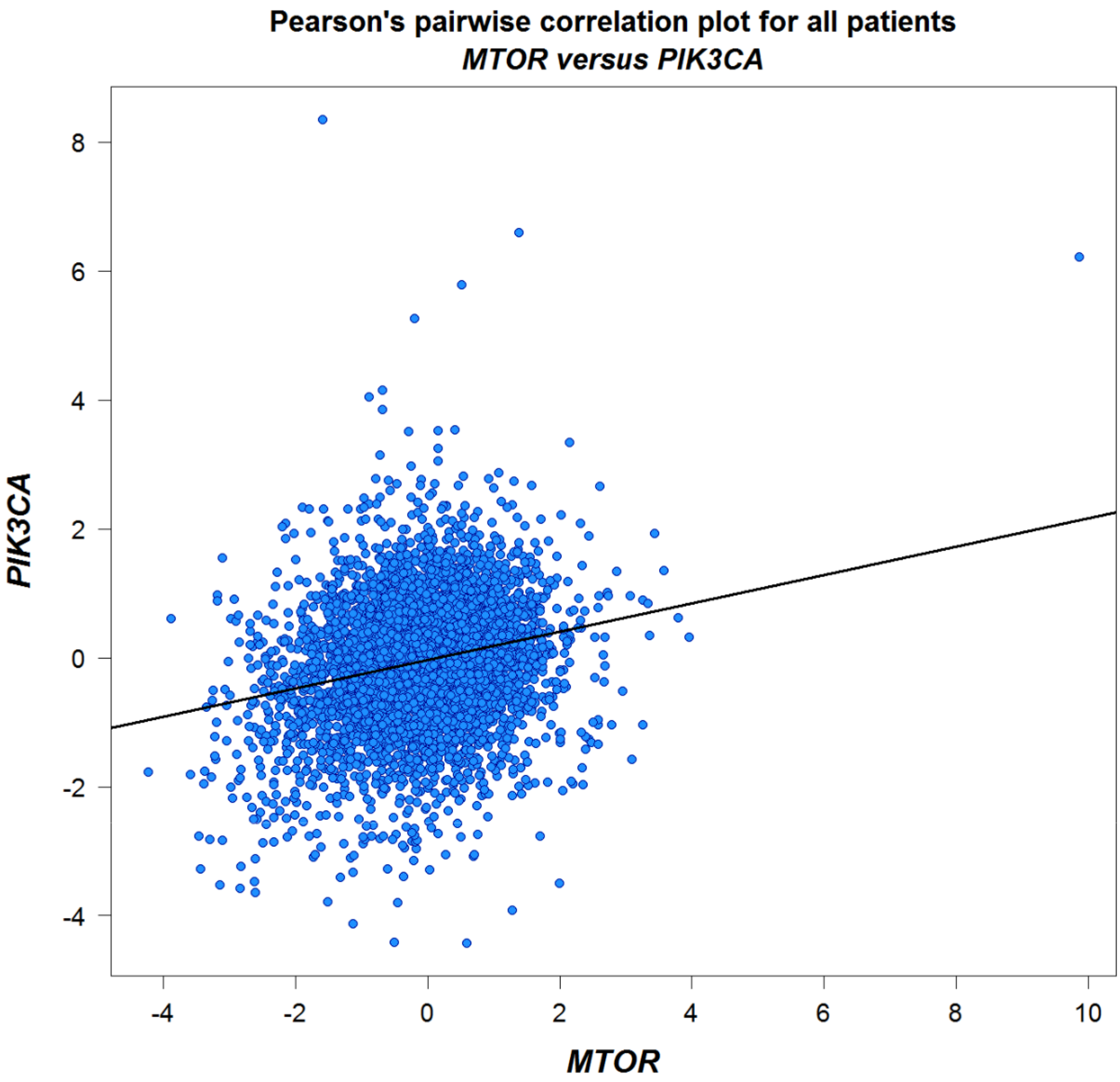

Figure S9. Correlation between the expression level of the *MTOR* and *PIK3CA* genes using Pearson's correlation coefficient in breast cancer- analysis the data available on international database TCGA (web source the bc-GenExMiner).

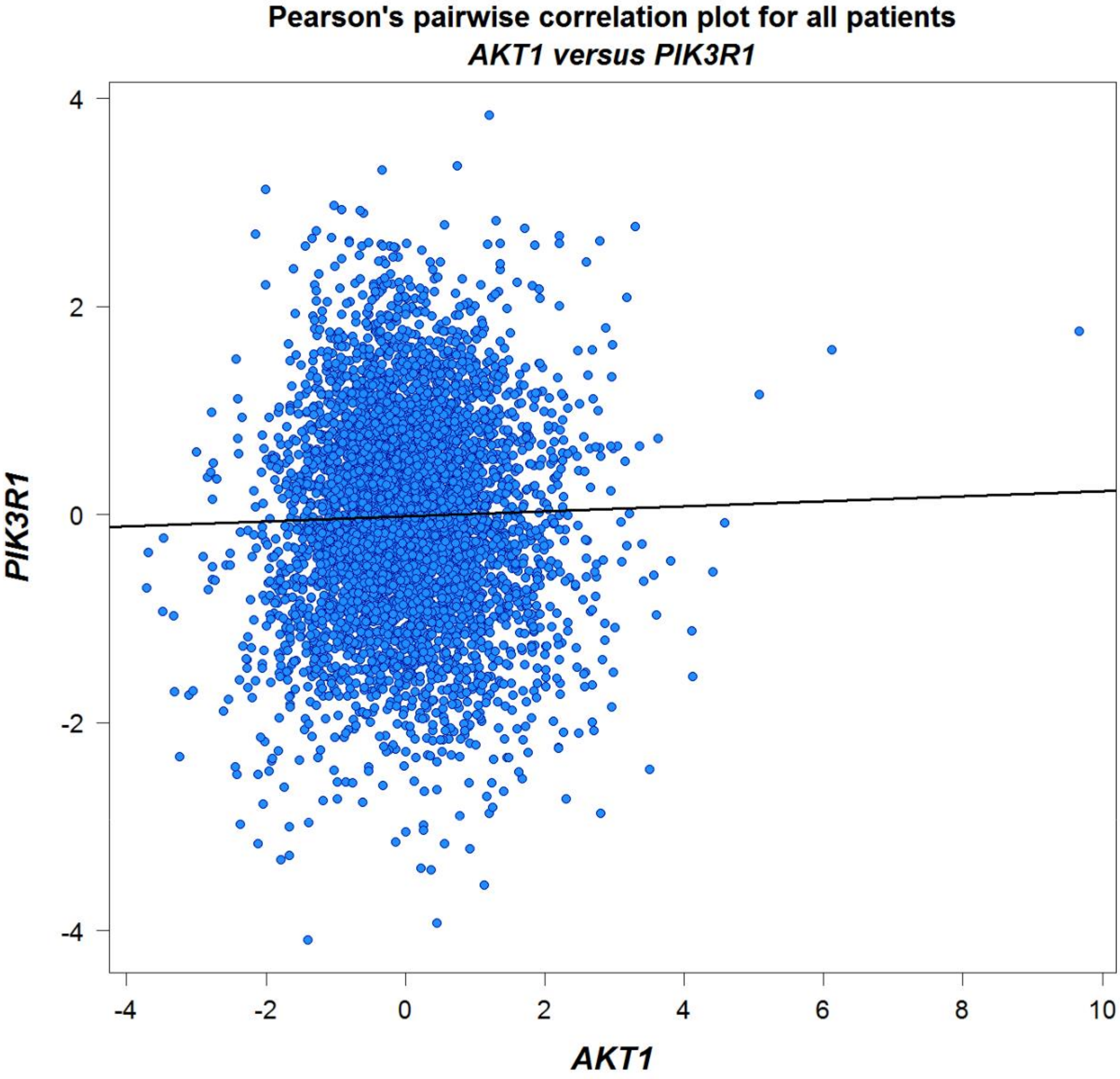

Figure S10. Correlation between the expression level of the *AKT1* and *PIK3R1*genes using Pearson's correlation coefficient in breast cancer- analysis the data available on international database TCGA (web source the bc-GenExMiner).

International Journal of *Molecular Sciences*

MDPI

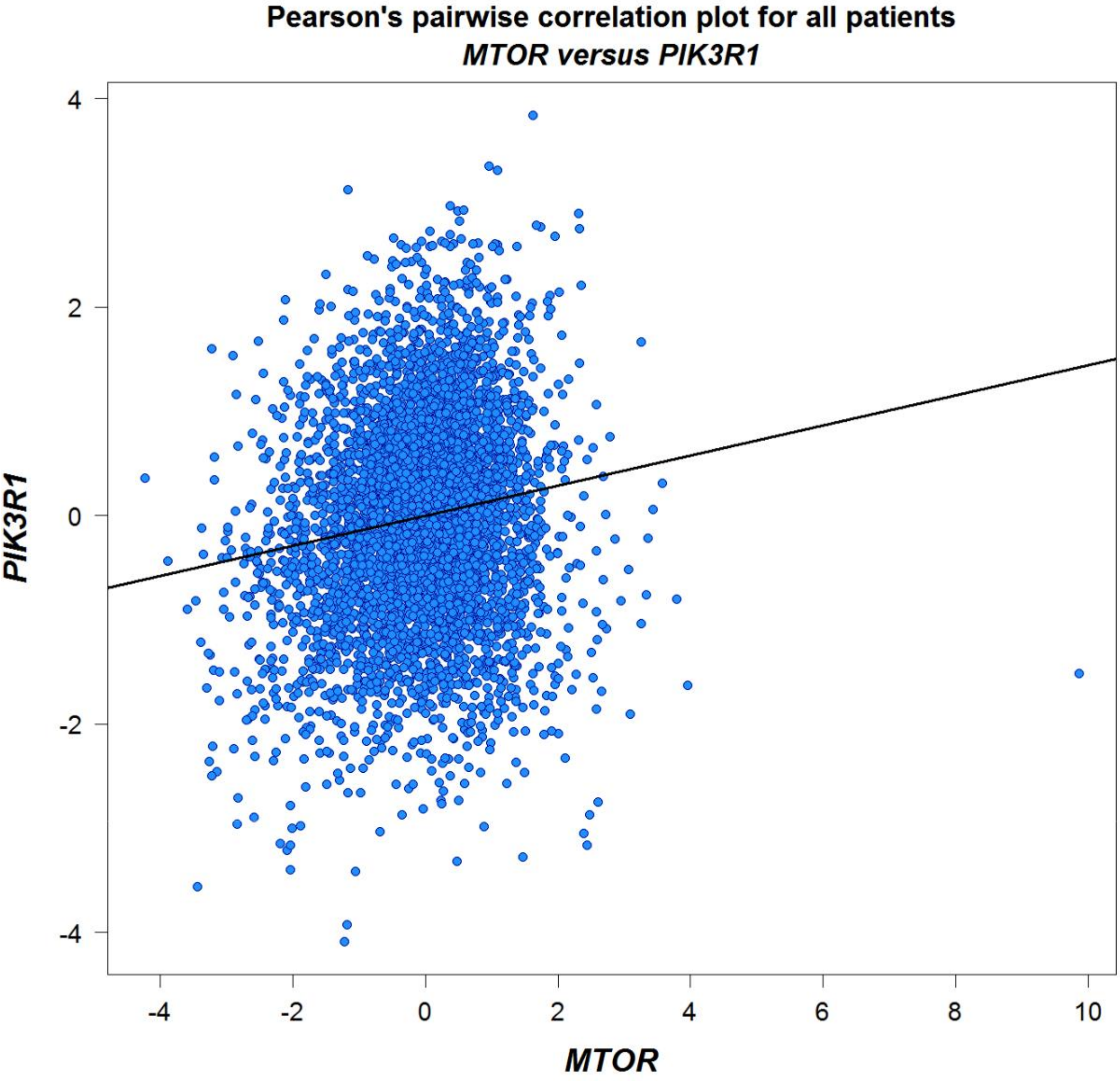

Figure S11. Correlation between the expression level of the *MTOR* and *PIK3R1* genes using Pearson's correlation coefficient in breast cancer- analysis the data available on international database TCGA (web source the bc-GenExMiner).

International Journal of *Molecular Sciences*

MDPI

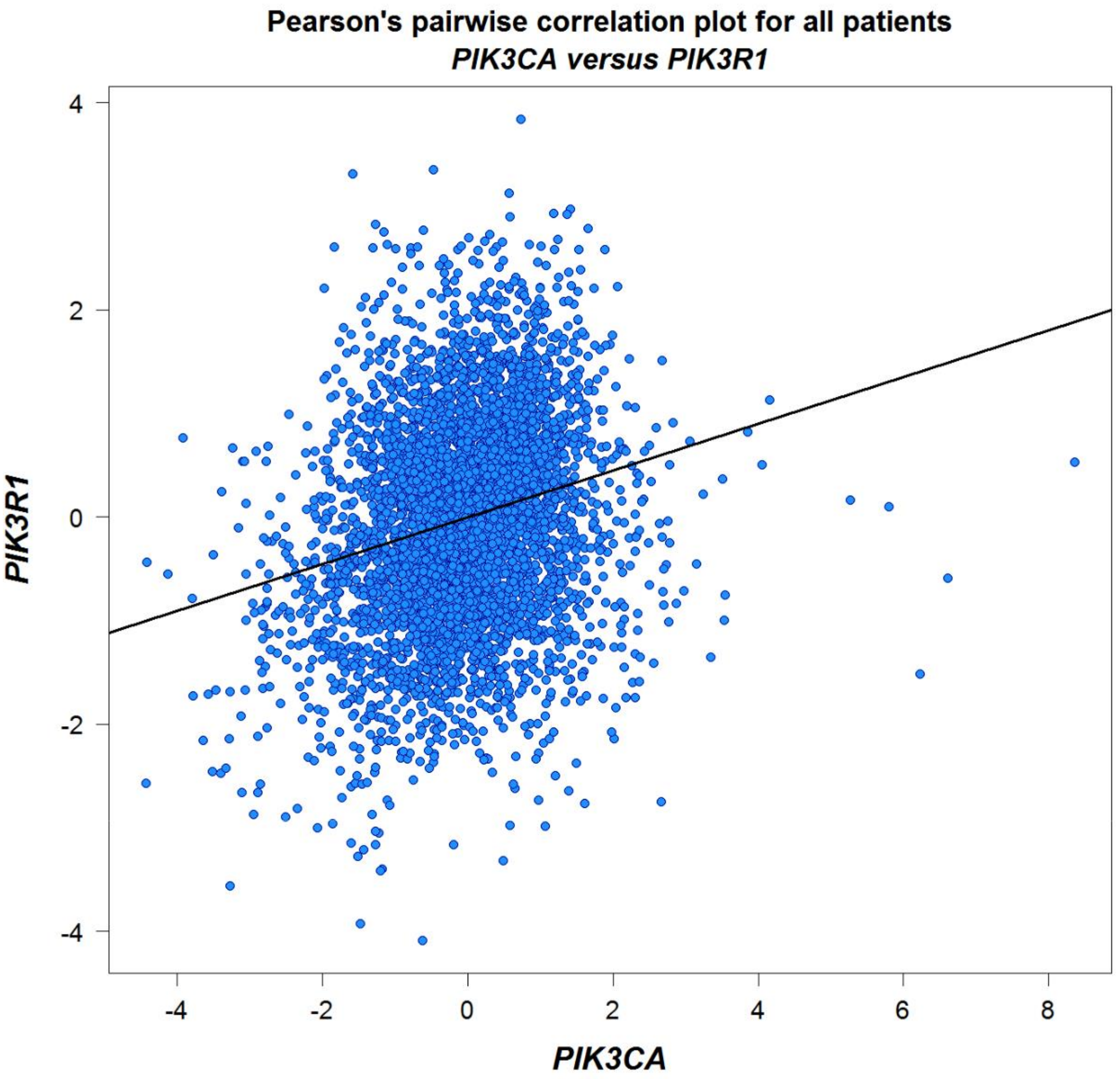

Figure S12. Correlation between the expression level of the *PIK3CA* and *PIK3R1*genes using Pearson's correlation coefficient in breast cancer- analysis the data available on international database TCGA (web source the bc-GenExMiner).

International Journal of *Molecular Sciences*

MDPI

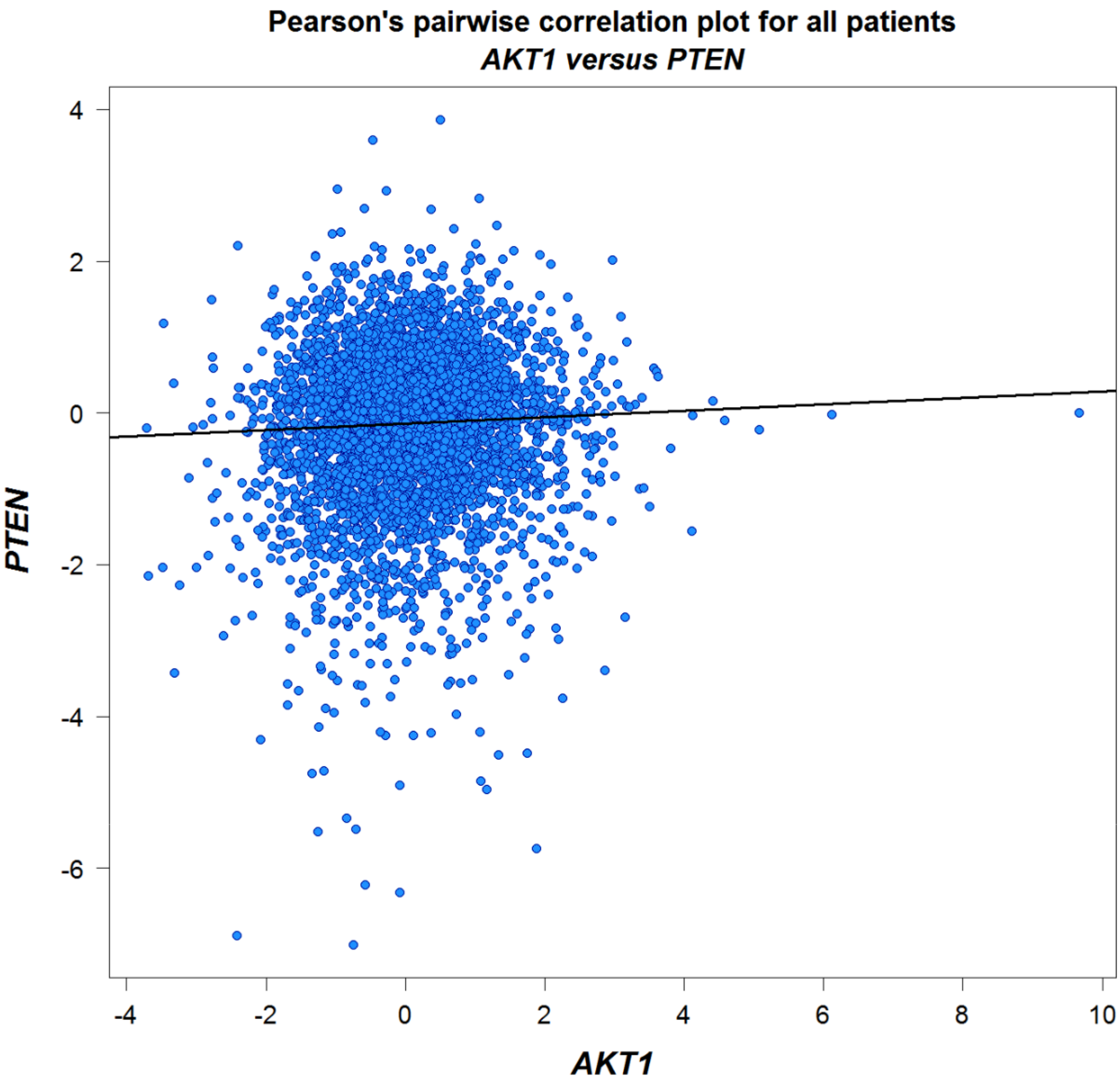

Figure S13. Correlation between the expression level of the *AKT1* and *PTEN* genes using Pearson's correlation coefficient in breast cancer- analysis the data available on international database TCGA (web source the bc-GenExMiner).

International Journal of *Molecular Sciences*

MDPI

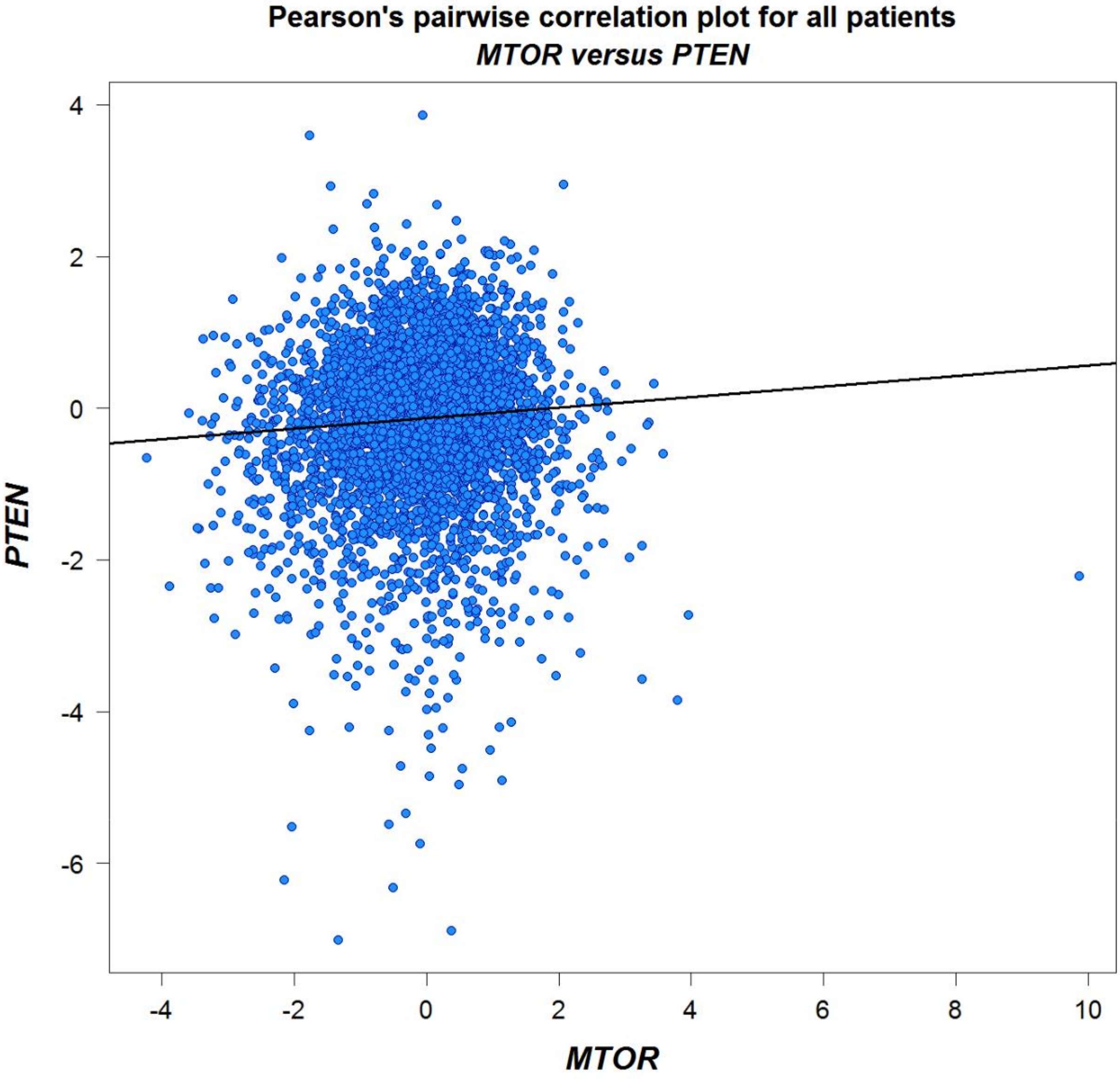

Figure S14. Correlation between the expression level of the *MTOR* and *PTEN* genes using Pearson's correlation coefficient in breast cancer- analysis the data available on international database TCGA (web source the bc-GenExMiner).

International Journal of Molecular Sciences

MDPI

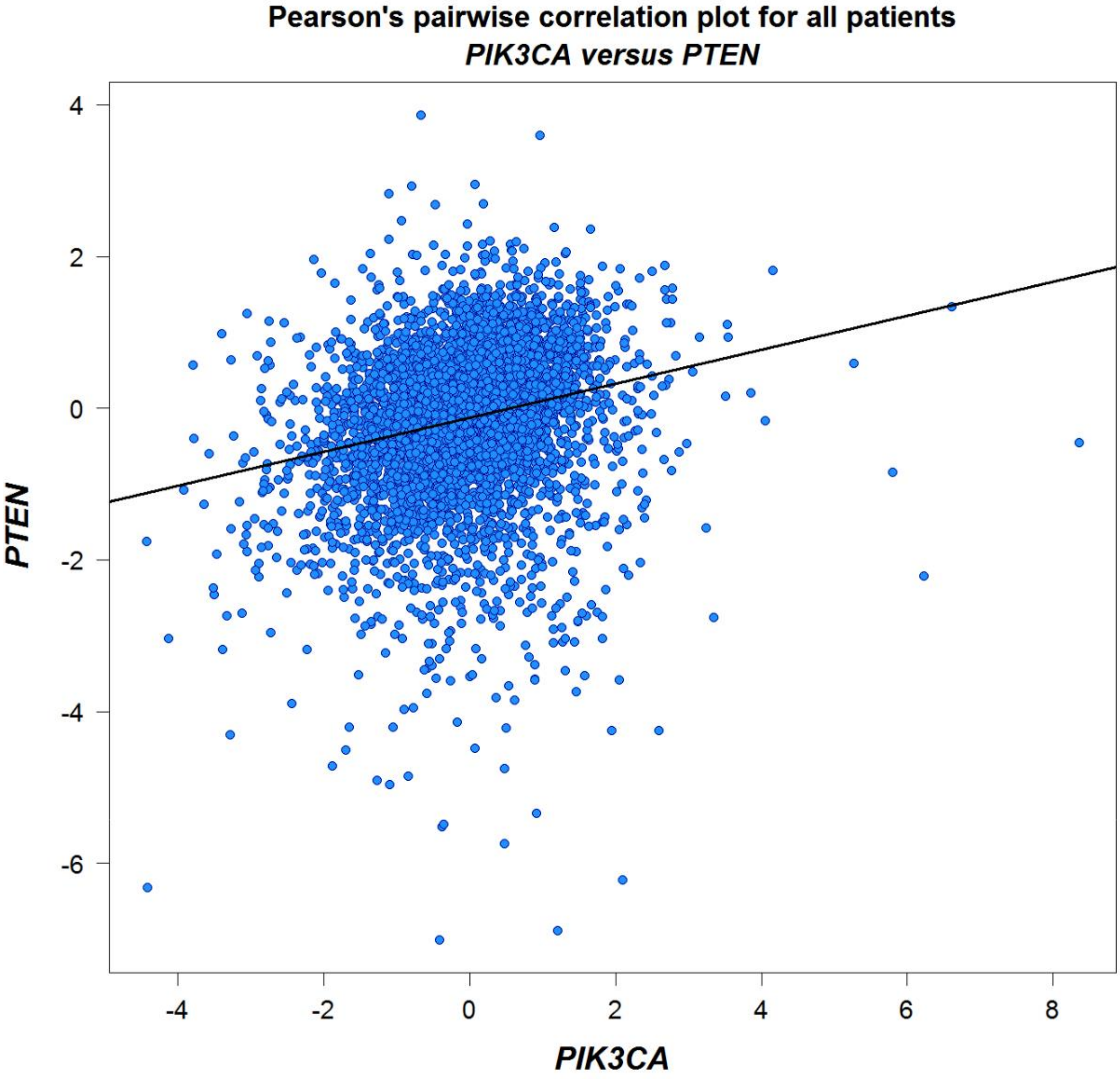

Figure S15. Correlation between the expression level of the *PIK3CA* and *PTEN* genes using Pearson's correlation coefficient in breast cancer- analysis the data available on international database TCGA (web source the bc-GenExMiner).

International Journal of *Molecular Sciences*

MDPI

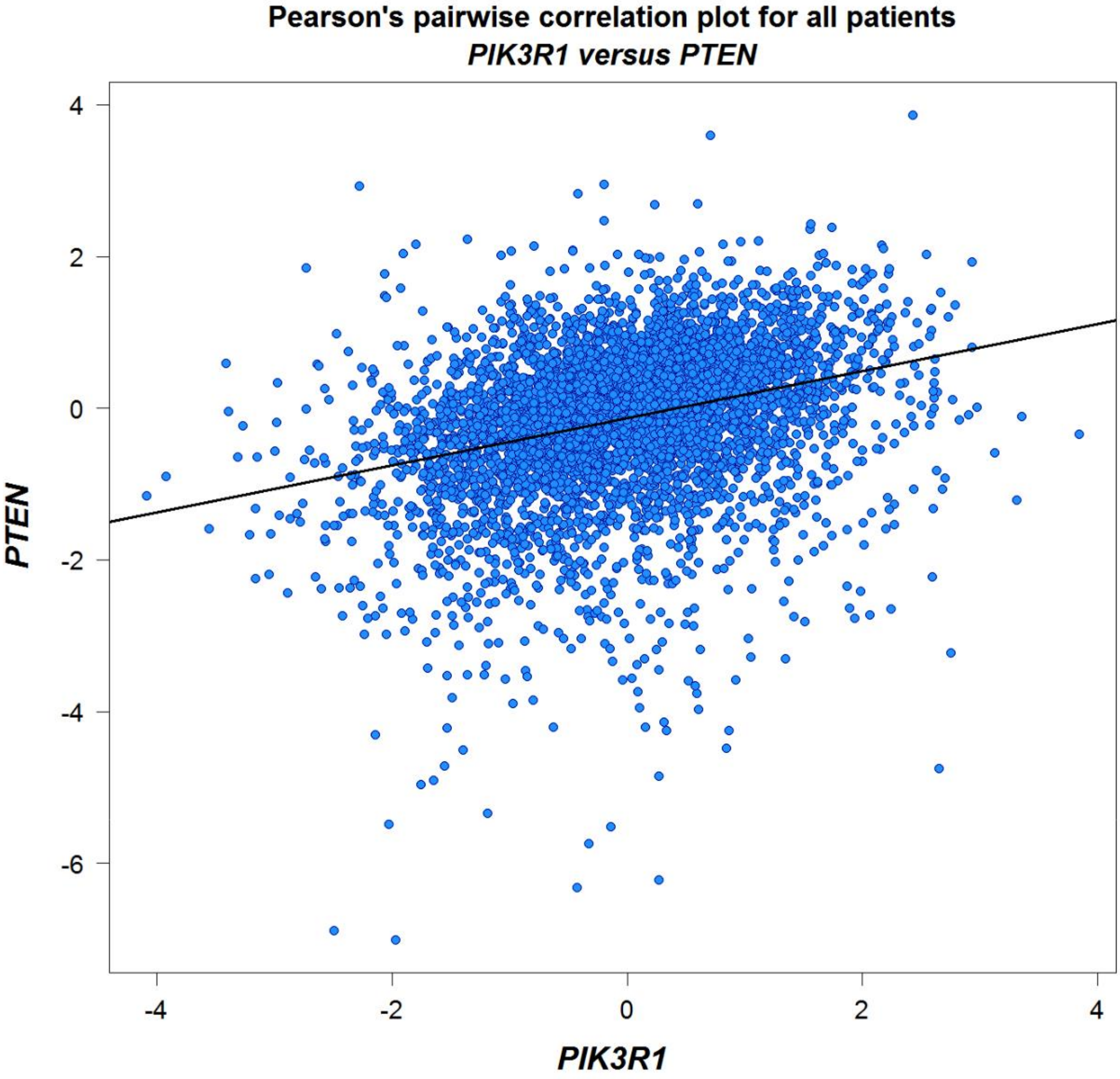

Figure S16. Correlation between the expression level of the *PIK3R1* and *PTEN* genes using Pearson's correlation coefficient in breast cancer- analysis the data available on international database TCGA (web source the bc-GenExMiner).

Table 11S. Correlation between expression level of the studied genes using Spearman's rank correlation coefficient in the entire studied group - the detailed statistical analysis. *p<0.05.

Genes	Correlation rang Spearmana					
	LogRQ *AKT1*	LogRQ *mTOR*	LogRQ *PIK3CA1*	LogRQ *PIK3CA2*	LogRQ *PIK3R1*	LogRQ *PTEN*
LogRQ *AKT1*	1,000000	0,874286	0,734796	0,316263	0,411245	0,759100
LogRQ *mTOR*	0,874286	1,000000	0,649144	0,270356	0,357599	0,714190
LogRQ *PIK3CA1*	0,734796	0,649144	1,000000	0,674913	0,651939	0,796224
LogRQ *PIK3CA2*	0,316263	0,270356	0,674913	1,000000	0,565189	0,638441
LogRQ *PIK3R1*	0,411245	0,357599	0,651939	0,565189	1,000000	0,575986
LogRQ *PTEN*	0,759100	0,714190	0,796224	0,638441	0,575986	1,000000

Table 12S. Correlation between expression level of the studied genes using Spearman's rank correlation coefficient in the group of patients without mutations - the detailed statistical analysis. *p<0.05.

Genes	Correlation rang Spearmana					
	LogRQ *AKT1*	LogRQ *mTOR*	LogRQ *PIK3CA1*	LogRQ *PIK3CA2*	LogRQ *PIK3R1*	LogRQ *PTEN*
LogRQ *AKT1*	1,000000	0,880566	0,792997	0,429143	0,386366	0,791224
LogRQ *mTOR*	0,880566	1,000000	0,589572	0,309852	0,282625	0,689575
LogRQ *PIK3CA1*	0,792997	0,589572	1,000000	0,701478	0,650420	0,760784
LogRQ *PIK3CA2*	0,429143	0,309852	0,701478	1,000000	0,681869	0,629811
LogRQ *PIK3R1*	0,386366	0,282625	0,650420	0,681869	1,000000	0,603020
LogRQ *PTEN*	0,791224	0,689575	0,760784	0,629811	0,603020	1,000000

International Journal of *Molecular Sciences*

MDPI

Table 13S. Correlation between expression level of the studied genes using Spearman's rank correlation coefficient in the group of patients with mutations - the detailed statistical analysis. *p<0.05.

Genes	**Correlation rang Spearmana**					
	LogRQ *AKT1*	LogRQ *mTOR*	LogRQ *PIK3CA1*	LogRQ *PIK3CA2*	LogRQ *PIK3R1*	LogRQ *PTEN*
LogRQ *AKT1*	1,000000	0,846154	0,441758	-0,265734	0,388235	0,564706
LogRQ *mTOR*	0,846154	1,000000	0,573427	-0,209091	0,437363	0,573626
LogRQ *PIK3CA1*	0,441758	0,573427	1,000000	0,356643	0,556044	0,591209
LogRQ *PIK3CA2*	-0,265734	-0,209091	0,356643	1,000000	0,104895	0,517483
LogRQ *PIK3R1*	0,388235	0,437363	0,556044	0,104895	1,000000	0,391176
LogRQ *PTEN*	0,564706	0,573626	0,591209	0,517483	0,391176	1,000000
